# Strategies for Enhancing Energy-Level Matching in Perovskite Solar Cells: An Energy Flow Perspective

**DOI:** 10.1007/s40820-025-01815-z

**Published:** 2025-06-24

**Authors:** Xiaorong Shi, Kui Xu, Yiyue He, Zhaogang Peng, Xiangrui Meng, Fayi Wan, Yu Zhang, Qingxun Guo, Yonghua Chen

**Affiliations:** State Key Laboratory of Flexible Electronics (LoFE) & Institute of Advanced Materials (IAM), School of Flexible Electronics (Future Technologies), Nanjing Tech University (NanjingTech), 30 South Puzhu Road, Nanjing, 211816 People’s Republic of China

**Keywords:** Perovskite, Energy loss, Energy-level alignment, Energy transfer, Charge carrier dynamics, HTMs, ETMs

## Abstract

Metal halide perovskites, owing to their remarkable optoelectronic properties and broad application prospects, have emerged as a research hotspot in materials science and photovoltaics. In addressing challenges related to energy loss, photoelectric conversion efficiency, and operational stability in perovskite solar cells (PSCs), various strategies have been proposed, such as improving perovskite crystallization, developing tandem architectures, and advancing interfacial engineering. However, the specific impact of these approaches on internal energy transfer and conversion mechanisms within PSCs remains insufficiently understood. This review systematically examines the relationship between energy and perovskite materials throughout the photon absorption to charge carrier transport process, with particular focus on key strategies for minimizing energy losses and their underlying influence on energy-level alignment-especially in the electron transport layer and hole transport layer. It summarizes optimal absorption conditions and contributing factors during energy transfer, alongside representative case studies of high-performing systems. By elucidating these mechanisms, this work offers valuable theoretical insights for optimizing energy-level alignment, reducing energy dissipation, and guiding experimental design in PSCs research.

## Highlights

- Energy transfer is systematically reviewed as a guiding principle for current materials and optimization strategies in perovskite solar cells.
- Characteristic mechanisms are identified to classify energy-level optimization strategies into two categories.
- Performance-enhancement strategies for perovskite solar cells are analyzed from a quantum-level perspective.

## Graphical abstract

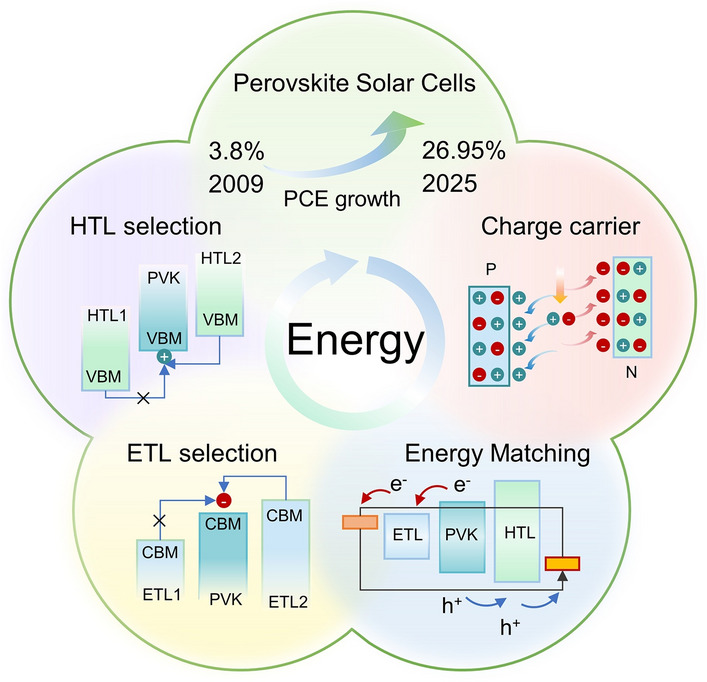

## Introduction

Perovskite materials, characterized by their ABX_3_-type crystal structure, have become a focus of active research due to their low cost, high efficiency, long lifespan, high flexibility, and versatility across various application scenarios [1]. Perovskites exhibit exceptional optical and electrical properties, such as excellent light absorption, high charge carrier mobility, long carrier diffusion length, ambipolar conductivity, and a direct bandgap, making perovskite solar cells (PSCs) a promising contender for next-generation photovoltaic technologies. Currently, PSCs have achieved a certified laboratory efficiency of 26.95%, which is comparable to leading photovoltaic materials, such as crystalline silicon cells and copper indium gallium selenide (CIGS) cells [2].

The mechanism of solar cells involves the conversion of solar energy into electrical energy through photovoltaic materials, where achieving higher power conversion efficiency (PCE) is crucial for reducing photovoltaic costs. However, energy losses occur during the conversion process, primarily including thermalization losses, below bandgap losses, optical losses, recombination losses, and spatial relaxation losses [3]. The ability of a material to absorb solar energy largely depends on its energy-level structure. The bandgap width determines the maximum wavelength for spectral absorption, while the number of electronic states in the valence and conduction bands influences the quantity and mobility of charge carriers.

**Fig. 1:**
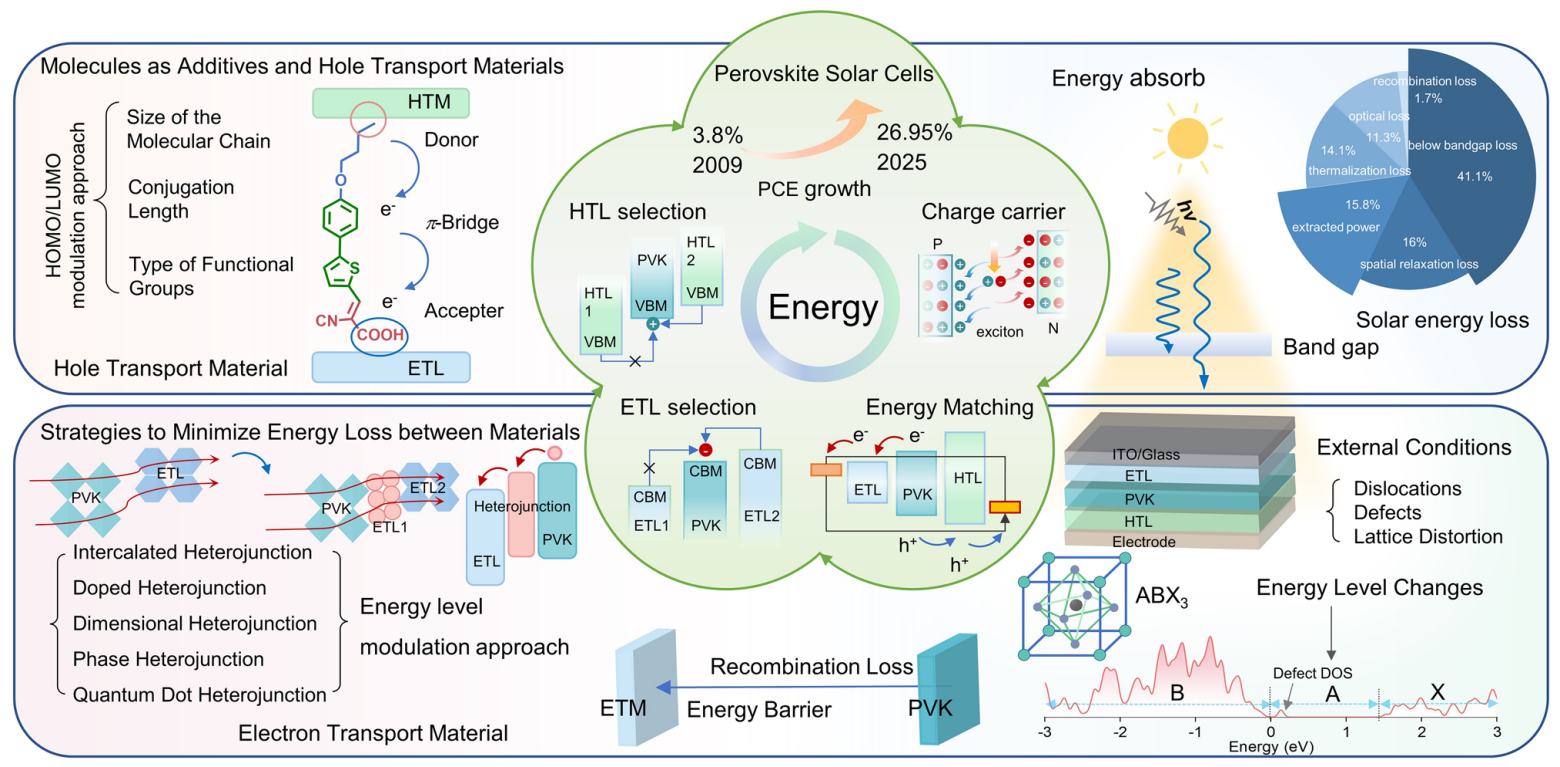
Schematic diagram of strategies to enhance energy-level alignment in perovskite materials based on energy transfer relationships [3]. This review focuses on material design, interface engineering, and energy-level modulation, analyzing the interrelationship between energy transfer and alignment strategies

To achieve optimal efficiency in solar cells, minimizing energy losses during the conversion and transport processes in perovskite devices is essential. Experimental studies have demonstrated that factors such as film morphology, device configuration, interface losses, and crystal quality are critical in determining PCE. Understanding the photophysical mechanisms of PSCs, as well as the pathways and losses involved in energy transfer between different materials, is key to improving the performance of PSCs. As shown in Fig. 1, this paper reviews the mechanisms of energy flow and loss during the photovoltaic conversion process, focusing on strategies to minimize energy losses in PSCs, and offers theoretical insights into the selection and optimization of organic–inorganic hybrid PSC materials.

## Working Principle of Solar Cells and Utilization of Photon Energy

### Photon and Energy Conversion in Solar Cells

**Fig. 2:**
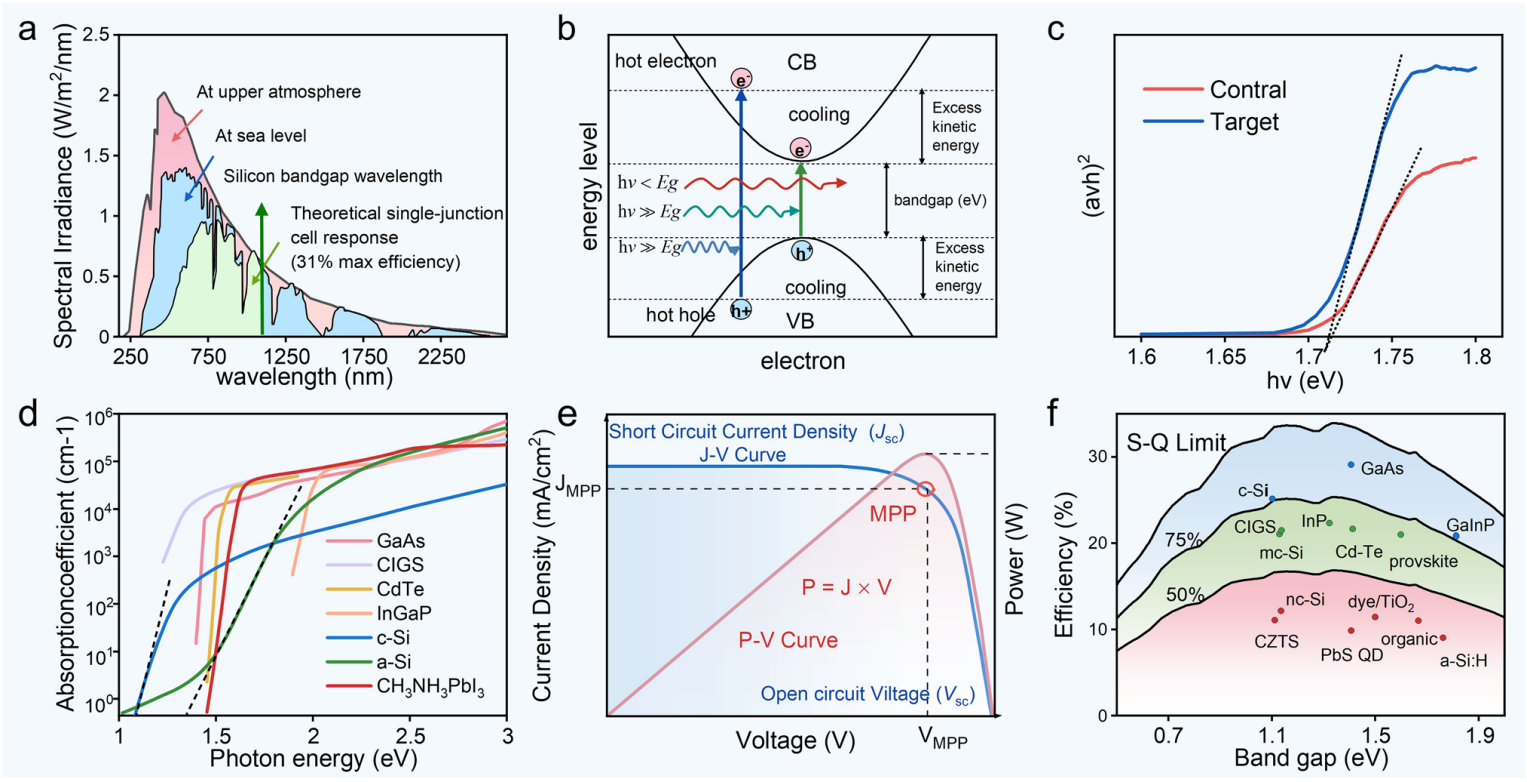
**a** Spectral power density of sunlight. Reproduced with permission from Ref. [4]. Copyright 2017, Science and Education Publishing. **b** Electron transitions induced by different light frequencies, the cooling process between energy bands, and the distribution of excess kinetic energy. Reproduced with permission from Ref. [5] Copyright 2022 Elsevier Inc. **c** Tauc comparison between CsPbI_3_ reference film and 0.6% CSE target film. Reproduced with permission from Ref. [6]. Copyright 2024, American Chemical Society. **d** Effective absorption coefficients of different photovoltaic materials [7]. **e** J-V and P–V characteristic curves of photovoltaic cells. **f** Correspondence curve between the bandgap of different photovoltaic materials and the S-Q limit efficiency and actual efficiency. Reproduced with permission from Ref. [8]. Copyright 2016, American Association for the Advancement of Science

Solar cells convert the energy of photons from sunlight into electrical energy, achieving photoelectric conversion. The energy of photons in sunlight originates from nuclear fusion reactions within the sun, where the energy generated by fusion is transmitted to the sun's surface through radiation and convection and is then dispersed outward in the form of electromagnetic waves. Light exhibits "wave-particle duality", displaying both particle and wave-like properties. The particle nature of light is evidenced by photons being able to propagate in a vacuum while carrying specific energy, whereas its wave-like nature is evident in phenomena such as diffraction and interference, with photons having a specific frequency and wavelength. The energy of a photon is directly proportional to its frequency, which can be calculated using Planck's equation *Ε* = *ℎν*. The photon energy of sunlight ranges from 0.5 to 3.5 eV, corresponding to wavelengths from 2.48 to 0.354 μm, covering infrared, visible, and ultraviolet light, which represents the overall solar spectrum. However, the effective absorption range for solar cell materials is generally between 1.1 and 3.5 eV (approximately 1.13 to 0.354 μm), mainly covering visible light and part of the near-infrared region, making it suitable for efficient energy utilization in photoelectric conversion. As sunlight passes through the atmosphere, certain specific wavelengths are absorbed by ozone, oxygen, and water vapor (Fig. 2a). In experimental settings, Air Mass X(AMX) spectral standards (X measured in multiples of atmospheric thickness) are often used to simulate natural light and evaluate the performance of photovoltaic devices.

### Photon Absorption Mechanism in Photovoltaic Semiconductor Materials

Upon absorbing photons of specific energies, electrons in photovoltaic semiconductors are excited to higher energy levels. Light absorption mechanisms can be classified into intrinsic absorption, exciton absorption, free carrier absorption, impurity absorption, and lattice vibration absorption, with intrinsic absorption being the dominant process in semiconductor materials [7]. As shown in Fig. 2b, when the photon energy is equal to or greater than the bandgap energy, electrons are excited across the bandgap into the conduction band, where they can move freely and contribute to current generation. This intrinsic absorption process is the fundamental mechanism underlying the photovoltaic effect and directly influences the photoelectric conversion efficiency of semiconductor materials [5].

Different semiconductor materials have different bandgap widths, and only photons with energy greater than or equal to the bandgap can be effectively absorbed. At the photon oscillation frequency *υ* = *E*_*g*_ /*ℎ* (where *E*_*g*_ is the bandgap and ℎ is Planck's constant), the absorption spectrum exhibits a steep absorption edge, while for *υ* < *E*_*g*_/*ℎ* (incident wavelength *λ* > 1.24/*E*_*g*_, with *E*_*g*_ in eV), the absorption coefficient is relatively low. The absorption coefficient is commonly used to indicate the material's effectiveness in intrinsic photon absorption. For instance, Fig. 2c shows the absorption of CsPbI_3_ films and target CsPbI_3_ films containing 0.6% CSE (1,2-bis(chlorodimethylsilyl)ethane) in the violet-visible spectrum (Tauc plot). The Cl⁻ groups in CSE effectively passivate Pb-related defects, suppress non-radiative recombination, and adjust the energy-level alignment of the PSCs to reduce open-circuit voltage (*V*_oc_) loss [6]. Both the control and target films have a bandgap of 1.71 eV, but the target films demonstrate a higher light absorption intensity, particularly in the 550–750 nm range. Figure 2d illustrates the spectral absorption curves of different photovoltaic materials [9], describing their light absorption capacity and response characteristics across various wavelengths, thereby allowing an evaluation of their potential for photoelectric conversion. The absorption coefficient of perovskite (CH_3_NH_3_PbI_3_) rises sharply near its bandgap, indicating its direct bandgap nature. Beyond the bandgap, the absorption coefficient quickly jumps to the order of 10^4^–10^5^ cm^−1^ and remains stable, significantly higher than that of conventional silicon-based materials such as c-Si and a-Si, suggesting that perovskites can achieve efficient light absorption even with extremely thin-film thicknesses. The high absorption coefficient and direct bandgap characteristics of perovskites make them highly advantageous for use in thin-film photovoltaic devices.

### Energy Distribution in Sunlight and Photon Absorption Efficiency

The total energy of sunlight under the Air Mass (AM) 1.5G standard is approximately 1000 W m^−2^, with photons carrying different energies distributed unevenly (Fig. 2a). In research, spectral photon flux ($\phi_{\lambda } { = }\frac{d\phi }{{d\lambda }}$, where *Φ* represents the number of photons in a unit wavelength range and *λ* represents the wavelength) is commonly used to describe the photon intensity within a specific wavelength range. The photon flux reaches its maximum around 500 nm. Theoretically, as the material's bandgap decreases, the range of absorbed photons expands, allowing more photons to be absorbed and increasing the light utilization efficiency. However, materials with smaller bandgaps may lead to thermalization losses, where electrons excited by high-energy photons quickly relax to the bottom of the conduction band (Fig. 2b) [5]. Additionally, an excessively small bandgap can cause a decrease in the open-circuit voltage (*V*_oc_) and affect the overall spectral utilization efficiency and stability. The J-V curve is commonly used to evaluate the performance of semiconductor materials in photovoltaic applications. Figure 2e shows the classic photovoltaic J-V relationship curve, where *V*_oc_ represents the open-circuit voltage (the voltage when current output is zero), and short-circuit current density (*J*_sc_) represents the short-circuit current density (the current density when the external circuit is shorted). On the J-V curve, there is a point where the product of current and voltage reaches its maximum, known as the maximum power point (MPP). The formula for calculating the power conversion efficiency (PCE) is:

1 $$ PCE = \frac{{V_{oc} \times J_{sc} \times FF}}{{P_{in} }} $$

2 $$ FF = \frac{MPP}{{V_{oc} \times J_{sc} }} $$

where P_in_ represents the incident light power, and FF is the fill factor. The fill factor is a critical parameter used to evaluate how closely the actual output power of a photovoltaic device approaches its theoretical maximum. A higher FF indicates lower non-radiative recombination and resistance losses within the device.

### Strategies for Enhancing Spectral Absorption

Solar cells only utilize a relatively small portion of the total photon energy from sunlight (about 1/3), because photons with energy below the optical bandgap undergo transmission, reflection, and scattering in the material, and their energy cannot be utilized. As a result, photovoltaic cells are limited by the Shockley-Queisser (S-Q) limit. Figure 2f shows the relationship between the bandgap of photovoltaic materials and the SQ limit [8]. Theoretically, the conversion efficiency limit for single-junction photovoltaic cells is 33.7%, which is the ideal bandgap (*E*_*g*_ = 1.4 eV) calculated for the AM 1.5G spectrum at 25 °C using the ASTM G173-03 standard, with a maximum power output of 337 W m^−2^ [10]. However, Wang et al. proposed that halide perovskites could theoretically break the material-level SQ limit through mechanisms such as hot carriers, multiple exciton generation, intermediate band gaps, and ferroelectricity [5]. Theoretically, single-junction solar cells can achieve efficiencies ranging from 44% to 55%, while multi-junction cells can reach a theoretical limit as high as 86%. However, a significant gap still exists between theoretical and practical efficiencies. In a recently published study, Liu et al. developed mixed self-assembled monolayers (NA-Me) by co-assembling [4-(3,6-dimethyl-9H-carbazol-9-yl)butyl]phosphonic acid (Me-4PACz) with the multi-aromatic carboxylic acid molecule 4,4′,4″- nitrilotribenzoic acid (NA) at the NiO/perovskite interface in inverted solar cells. This approach leverages intermolecular π–π interactions, suppressed interfacial nanovoids, homogenized hole transport, and passivated V_Pb_^2+^ defects, ultimately achieving an optimal efficiency of 26.69% [11], which is the highest efficiency of single-junction perovskite solar cells in the current published articles.

The below bandgap losses in perovskite solar cells account for 41.1% of the total energy loss in solar cells [3]. The excess kinetic energy from high-energy photons is released as heat through electron–phonon scattering, causing the cooling of hot electrons and holes [12]. To more effectively utilize a broader portion of the solar spectrum, Bell Labs proposed stacked solar cells in the twentieth century, which use different bandgaps to absorb light corresponding to different photon energies in series. For example, Liu et al. proposed a two-terminal monolithic perovskite-silicon tandem solar cell with LiF and ethylenediammonium diiodide (EDAI) bilayer passivation, achieving higher open-circuit voltage and fill factor, and achieving a tandem efficiency of 33.89% [13], LONGi achieved a high efficiency of 34.6% in 2025 using a tandem structure of perovskite and Si.

Beyond material design and tandem configurations, photothermal or up/down conversion represent additional strategies for enhancing light absorption. Photothermal hybrid systems reduce energy loss by recovering waste heat and converting it back into electrical energy. Up/down conversion transforms low-energy photons (via up-conversion) and high-energy photons (via down conversion) into photons that are absorbable within the material's bandgap, thereby expanding spectral utilization and increasing overall efficiency [5].

### Band Gap Advantage of Perovskites in Photovoltaic Materials

As shown in Fig. 2f, there is still a gap between the current experimental values of photovoltaic materials and the theoretical values derived from the bandgap. The ideal bandgap for solar cells is 1.4 eV [14], which corresponds to a critical photon wavelength of 886 nm (infrared light). The theoretical maximum efficiency of crystalline silicon solar cells with a 1.1 eV bandgap is 29.43% [15]. The high efficiency currently achieved is 27.3% by the back-contact crystalline silicon heterojunction solar cells developed by LONGi Green Energy [2]. The highest experimental efficiency for copper indium gallium selenide (CIGS) solar cells with a 1.15 eV bandgap is 23.6% [16]. GaAs solar cells with a 1.45 eV bandgap have achieved 29.1% efficiency in experiments (due to high production costs, they are typically used in aerospace applications) [2]. Perovskite solar cells, due to their low production cost and tunable bandgap, are considered an important development direction for the future.

The crystal structure of perovskite is ABX_3_, allowing for a variety of material combinations. The all-inorganic perovskite CsPbI_3_ with a 1.74 eV bandgap has achieved an experimental efficiency of 22.14% [17]. Organic–inorganic hybrid perovskites can adjust their bandgap by changing the composition. For example, the hybrid perovskite CH_3_NH_3_PbI_3_ (MAPbI_3_) with a 1.55 eV bandgap has achieved an experimental efficiency of 24.12% [18]. Among them, HC(NH_2_)_2_PbI_3_ (FAPbI_3_) (with a bandgap of 1.48 eV), which is closest to the ideal value, has achieved an experimental efficiency of 26.95% [2]. The excellent light absorption capability of perovskites gives them tremendous potential for future development in the photovoltaic field.

## Material Structure and Performance Optimization Strategies for Perovskite Solar Cells

### Intrinsic Relationship Between Perovskite Crystal Structure and Light Absorption Properties

**Fig. 3:**
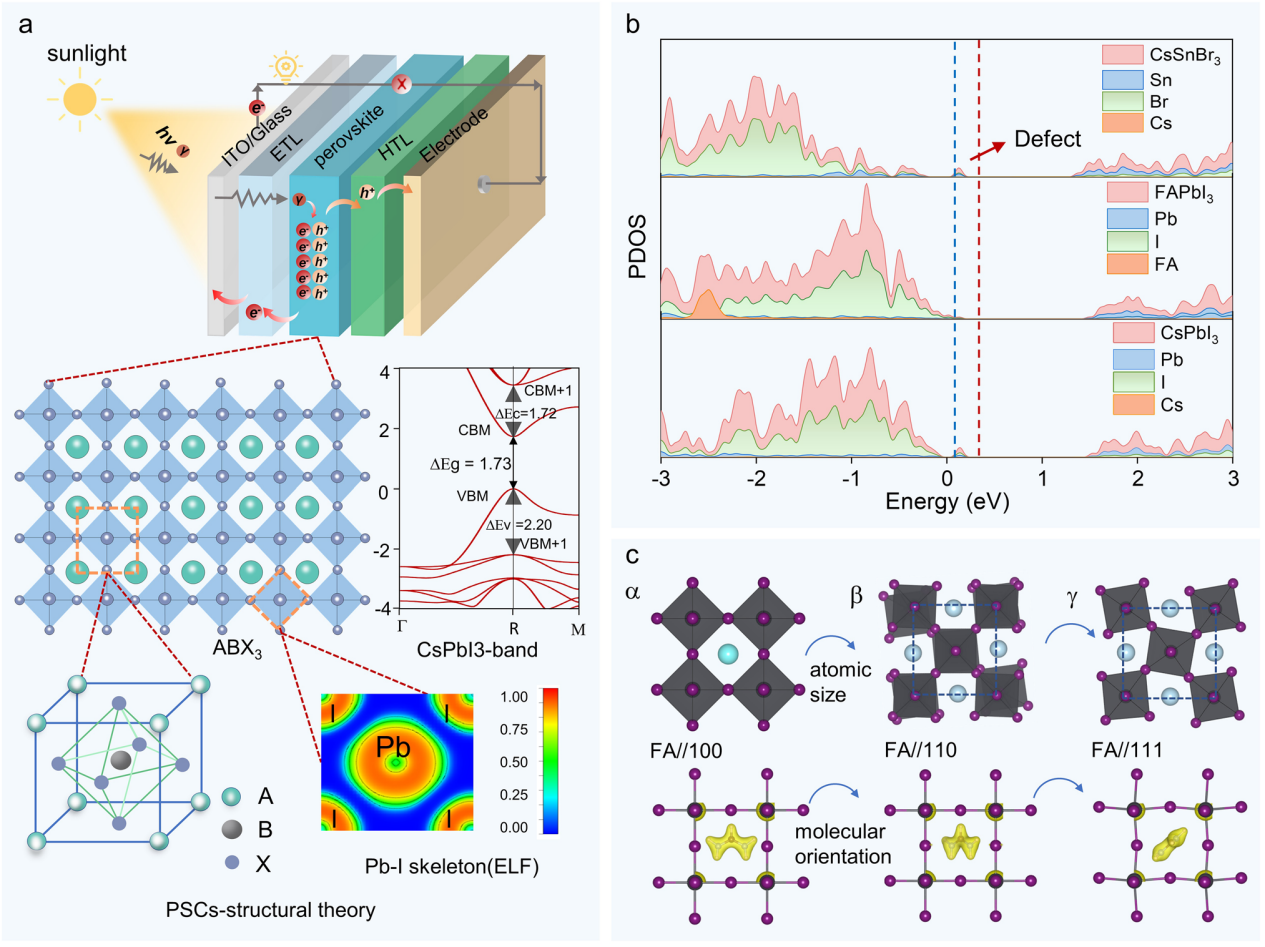
Theoretical Structure and Working Principle of Perovskite Solar Cells. **a** Photovoltaic conversion process of PSCs and the ABX_3_ crystal structure of perovskite, bandgap characteristics (Reproduced with permission from Ref. [21]. Copyright 2023, John Wiley and Sons) and bonding characteristics (electron localization function (ELF)) of PVK. **b** Density of states (DOS) of different types of perovskites. **c** Main phase transition factors of organic and inorganic perovskites

The photon energy utilization in perovskite depends on its unique material structure. As shown in Fig. 3a, metal halide perovskite is a crystalline material with an ABX_3_ structure, where A is a monovalent cation, B is a divalent cation, and X is an anion. The perovskite framework consists of corner-sharing [BX_6_]^−^ octahedra, with the B cation occupying the center of the octahedron and the X anion positioned at the vertices [19]. The redox interaction between the B-site and X-site is the basis of the photogenerated current in perovskite materials. The B–X bond exhibits both ionic and covalent characteristics, while the A-site cation, residing in the cavity formed by the [BX_6_] octahedra, interacts with the framework through van der Waals forces and hydrogen bonding (in the case of organic cations). Halide perovskites exhibit a direct bandgap at the R point in the ideal cubic phase, allowing electrons to transition from the valence band maximum to the conduction band minimum without phonon assistance. This results in a high absorption coefficient, which is beneficial for efficient photoelectric conversion. The valence band maximum (VBM) of halide perovskites is typically determined by the antibonding states between the s orbitals of the B-site metal and the *p* orbitals of the X-site halogen. The conduction band minimum (CBM) is composed of antibonding states between the *p* orbitals of the B-site metal and the *p* orbitals of the X-site halogen. The B-site cation dominates the lower conduction band position, while the X-site anion dominates the higher valence band position, as shown in Fig. 3b, the density of states (DOS) of CsPbI_3_, FAPbI_3_, and CsSnBr_3_, the electronic states at X in the valence band and those at B in the conduction band are occupied. Therefore, the choice of elements for B and X sites directly affects the bandgap size of the metal halide perovskite, thereby influencing light absorption.

Currently, common B-site cations include Pb^2+^, Sn^2+^, and Ge^2+^, while X-site anions include I^−^, Br^−^, or Cl^−^ [20]. The A-site cation is located in the cavities formed by multiple [BX_6_]^−^ octahedra. Common A-site cations include cesium (Cs^+^), methylammonium (MA^+^), and formamidinium (FA^+^). Due to their lower occupied energy levels, they generally exhibit less charge activity, as shown in Fig. 3b, the energy bands at the A-site are weakly occupied, and the substitution of FA^+^ and Cs^+^ has a minimal impact on the energy-level arrangement. The A-site cation plays a crucial role in structural stability and charge balance. The size of the A-site cation and its interaction with the [BX_6_]^−^ octahedra significantly influence the octahedral crystal arrangement, and therefore also exert a regulatory effect on the energy levels. At the same time, perovskites can adjust the arrangement of electronic energy levels or bandgap through the substitution of site atoms, achieving matched energy levels that align with the solar absorption spectrum.

### Impact of Crystal Structure Changes on Energy Utilization

According to the Goldschmidt criterion, the ionic radii of the A, B, and X-site cations must satisfy the ion packing arrangement assessed by the tolerance factor (*T*).

3 $$ T = R_{A} + R_{X} /\sqrt {2} (R_{B} + R_{X} ) $$

With *R*_*A*,_ *R*_*B,*_, and *R*_*X*_ representing the ionic radii of the A, B, and X-site cations, respectively [22, 23]. The ideal perovskite compound adopts a cubic close-packed structure with *Τ* = 1. When the ratio of ionic radii deviates from the ideal value (*Τ* ≠ 1), geometric strain and crystal distortion occur, leading to structural instability that can cause changes in the arrangement of electronic states, which in turn affects the device's lifetime and light absorption [24].

The size of the A-site cation in perovskite is limited to 2.6 Å, and the tolerance factor (*T*) for stable perovskites must be between 0.8 and 1.0. Inorganic perovskites exhibit better thermal and chemical stability, and the A-site organic cations that meet the conditions include Cs^+^, Rb^+^, and K^+^. The ideal crystal structure of halide perovskites is the cubic phase (*α*). In CsBX_3_, the inorganic cation Cs^+^, due to its smaller occupancy ratio, causes the corner-sharing [BX_6_]^−^ octahedra to easily tilt, leading to the formation of the tetragonal phase (*β*) and orthorhombic phase (*γ*), which alters the energy-level arrangement and thus affects light absorption. For example, in CsPbI_3_, the structure transitions from *α* to *β* and then to *γ,* with the Pb-I-Pb bond angle decreasing from 180° to 163° and then to 153°, and the bandgap increasing from 1.36 to 1.58 eV and then to 1.77 eV (Fig. 3c) [25, 26]. Typically, larger A-site cations tend to increase the B-X atomic distance, reducing orbital overlap and widening the bandgap. However, non-rigid organic cations (FA⁺, MA⁺), due to factors like asymmetric forces and hydrogen bonding, can alter the tilt angle of the perovskite crystal structure, thereby changing the arrangement of energy levels. For example, in MAPbI_3_, the different orientations of MA^+^ and the N–H bonds lead to varying degrees of tilting in the perovskite structure, causing changes in the energy levels. By controlling the molecular orientation, the arrangement of energy levels can be regulated (Fig. 3c) [27, 28]. Organic cations that meet the ion size requirements primarily regulate the electronic energy-level arrangement of perovskites by fine-tuning the interaction forces between the A-site cations and the [BX_6_]^−^ octahedra, as well as optimizing the interlayer spacing. Cations larger than the limiting radius can cause layer separation in the crystal structure, leading to the decomposition of the perovskite into a 3D form, which is unfavorable for perovskite formation. However, in 2016, Hu et al. discovered that by coating the surface of 3D perovskite with mixtures of MAI and large-volume molecules such as n-butylammonium iodide (BAI), a layered perovskite structure was formed, which improved the efficiency and performance of the solar cells [29]. Ma et al. also demonstrated that 2D/3D hybrid perovskites do not hinder electron transport and can passivate the 3D perovskite, resulting in higher PCE and better water stability [30]. Meanwhile, Mali et al. achieved an efficiency of 21.5% with the *β*-CsPbI_3_ and *γ*-CsPbI_3_ phase heterojunction all-inorganic perovskite solar cells in 2023. Therefore, effectively utilizing ion size to dope the ABX_3_ structure is an effective way to optimize perovskites [31].

Furthermore, vacancies and misalignments at the A, B, and X sites are key factors affecting the quality of perovskite films, this is particularly critical in non-radiative recombination processes. As shown in Fig. 3b, density functional theory (DFT) calculations indicate that X vacancy defects on the ABX_3_ surface introduce defect states. The defect region allows electrons to become unstable in orbital overhangs, causing the energy levels of the electronic states to appear in the forbidden band region. These energy states can capture transition electrons, leading to recombination losses and ion migration, which in turn reduce device performance. During the fabrication of perovskite solar cells, factors such as lattice constant mismatch, unsaturated chemical bonding, process residues, and differences in thermal expansion coefficients contribute to the formation of these defects. Passivating these defects by employing ions or functional groups to bind the dangling electrons is an effective approach, which reduces the likelihood of energy falling into trap states during transfer, thereby minimizing energy losses.

### Structure of Perovskite Solar Cells and Energy Transfer Mechanisms

As shown in Fig. 3a, perovskite ABX_3_ crystalline materials continuously absorb photons with energy greater than the bandgap energy, exciting electrons out of their energy orbitals to form electron–hole pairs. The directional flow of electrons generates photocurrent. When conductive electrodes and wires are incorporated, they form a conductive circuit, which is the most fundamental circuit structure of a solar cell. The generated electron–hole pairs can naturally diffuse to achieve electron–hole separation. However, in perovskites, the photogenerated electron–hole pairs lack directionality, making them prone to secondary recombination, which leads to energy loss. Therefore, when fabricating solar cells, it is crucial to promptly extract the electron–hole pairs in both directions to minimize energy loss. Although n-type and p-type perovskite tandem cells can promote electron transfer, research tends to focus on introducing electron transport layers (ETLs) and hole transport layers (HTLs) to accelerate electron–hole separation [32]. Introducing ETLs and HTLs can effectively improve the generation rate and mobility of photogenerated carriers, thereby enhancing the open-circuit voltage and overall energy conversion efficiency of the photocurrent.

Currently, the structure of perovskite solar cell devices is mainly divided into five parts (five-layer structure) (Fig. 3a), including conductive glass (such as ITO or FTO), electron transport layer, perovskite light-absorbing layer, hole transport layer, and conductive metal layer [33]. In this structure, electrons move from the perovskite layer into the electron transport layer and ultimately reach the electrode, while the holes generated by the perovskite move through the hole transport layer to the corresponding electrode. Electrons flow out of the electrode, pass through the external circuit, and finally recombine with the holes at the hole-electrode, forming a complete current loop. When sunlight radiation strikes the perovskite device, perovskite absorbs most of the light waves and generates charge carriers. Since the orbital energy levels of the materials are at the same energy level, electrons are more likely to transfer. However, due to the differences in material properties and interface effects between the perovskite and transport layers, energy barriers may exist, leading to interruptions in electron transport and requiring extra energy. Therefore, the bandgap matching and material selection between layers are crucial [34]. In experiments, heterojunctions or molecular binders are commonly used as bridges for energy barriers, thereby reducing energy loss in electron transport and improving solar energy conversion efficiency. Optimizing the matching of these materials to maximize photon absorption and extraction efficiently has become one of the core challenges in the design and fabrication of PSCs [35].

## Design of Electron Transport Layer

### Main Functions and Types of Electron Transport Layer

The core function of the electron transport layer (ETL) is to facilitate the transfer of electrons and suppress the reverse flow of holes. Ideal ETL materials should possess high electron mobility, excellent thermal and water stability, and be able to effectively block hole back-injection, while achieving good adhesion and interface energy-level alignment with the adjacent layers. The selection of ETL materials directly affects the efficiency and the stability and consistency of the output current of the solar cell. Suitable organic, inorganic, or organic–inorganic hybrid materials can be chosen as electron transport layers. Organic materials offer structural diversity, controllable energy-level alignment, abundant raw materials, and are suitable for large-scale manufacturing and flexible applications [36, 37]. In contrast, inorganic materials have simple structures, low preparation costs, and exhibit superior carrier transport performance and long-term stability, particularly under varying environmental conditions [38, 39]. This gives inorganic materials a significant advantage in terms of the device's service life.

To date, numerous inorganic electron transport materials have been reported and applied, including metal oxides, metal oxide composites, and metal oxide nanoparticles. Transition metal oxides are among the primary choices for electron transport materials. Compared to pure metals, which lack a bandgap and thus cannot effectively distinguish between charge carriers, transition metal oxides possess discrete conduction and valence bands. This allows for efficient charge transport while enabling selective conduction pathways for electrons and holes [37]. Common electron transport materials include TiO_2_, ZnO, SnO_2_, CdS, C_60_, Bathocuproine (BCP), and Phenyl-C61-butyric acid methyl ester (PCBM) [40–52].

### Energy-Level Matching Requirements for ETL and the Impact of Common Materials on Energy Transfer

**Fig. 4:**
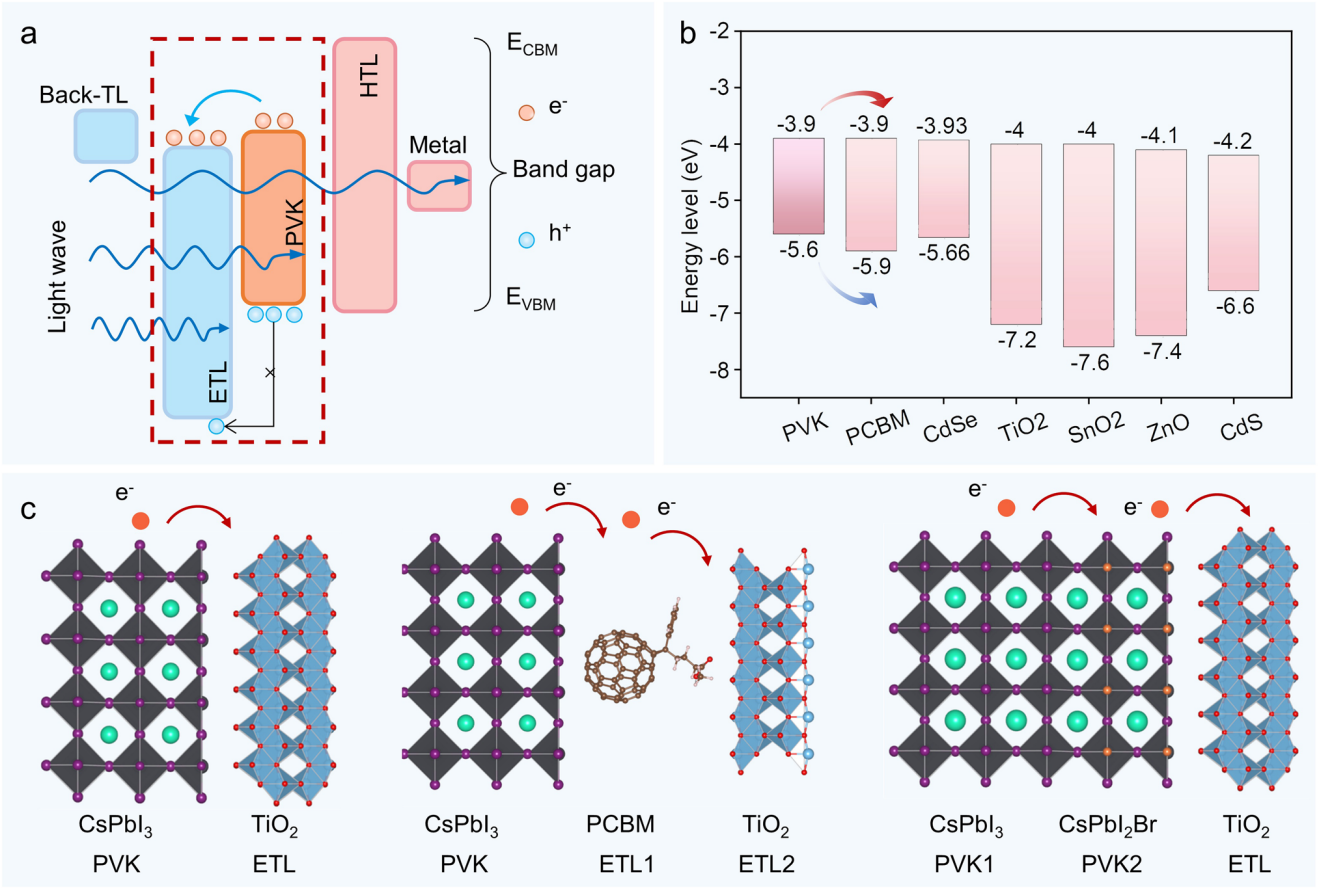
Electronic transport layer theory. **a** Schematic diagram of the electron transport principle. **b** Energy-level alignment of common electron transport materials. **c** General structural model of perovskite to electron transport layer

As shown in Fig. 4a, after the electron and hole separate upon photoexcitation and transition to the conduction band, electron transport occurs between the conduction bands of different layers. The lower energy difference between the electron transport material (ETM) and the conduction band minimum of the perovskite reduces the energy barrier for electron transfer to the ETL, enhancing electron migration to the ETL and minimizing electron–hole recombination at the interface. The larger energy difference between the ETM and the valence band maximum of the perovskite effectively blocks hole injection, thereby reducing electron–hole recombination within the ETL [53].

In the energy-level alignment of common electron transport materials (ETMs) (Fig. 4b), the zinc blende structure of CdS and perovskite have similar energy-level alignments, which allows them to be used for electron and hole transport. However, CdS has poor selectivity for electrons and holes, and thus is often used in passivation layers or multi-junction structures to improve electron injection efficiency at the perovskite interface [48, 49, 54, 55]. In contrast, materials such as ZnO, TiO_2_, and SnO_2_ possess excellent energy-level alignment and wide-bandgap properties. However, ZnO's high exciton binding energy enables efficient exciton recombination at room temperature, resulting in intense luminescence. This characteristic makes it a promising material for applications in UV lasers and light emitting diodes (LEDs) [44, 56–58]. PCBM, as a derivative of C_60_, has carboxyl group modifications that lower the conduction band minimum of C_60_ making it more effective at receiving photogenerated electrons and effectively blocking hole injection [34, 51, 59]. Among the various ETL materials, TiO_2_ has high chemical stability and low-cost preparation methods, while SnO_2_ exhibits high electron mobility and good thermal stability, making them commonly used ETMs in experiments [40, 41, 60–63]. How to fully utilize the advantages of each ETM to fabricate higher-performance perovskite solar cells is the focus of our research.

### Strategies for Optimizing Energy-Level Matching for Electron Transport in ETL

The aforementioned materials inherently possess different advantageous characteristics, but the issues of intrinsic defect density and energy-level mismatch cannot be ignored [64]. Research continues to explore ways to further optimize their thermal stability, corrosion resistance, and ease of preparation [65–68]. Reducing energy loss during electron transport is key to optimization strategies. Currently, a large number of studies have reported methods to regulate interface effects and reduce energy loss, which can be broadly divided into two categories: heterojunction design and additive engineering [42]. Heterojunction design, in most cases, does not significantly alter the physical properties of the original materials but rather uses their inherent characteristics to achieve energy-level tuning [69]. Whereas additive engineering involves the introduction of organic molecules or solvents to modify the physical and chemical properties of the interface, affecting the energy-level alignment between the perovskite layer and ETL [70, 71]. Furthermore, heterojunction and additive engineering can also improve the transport layer interface morphology, passivate the surface to prevent degradation or erosion, and enhance device stability. These engineering strategies can modify the transport layer by inducing strain in its lattice or molecular morphology and passivating defects such as vacancies. This results in more uniform and stable materials, thereby improving device performance. This chapter focuses only on heterojunction engineering, while additives are discussed in the hole transport layer.

**Fig. 5:**
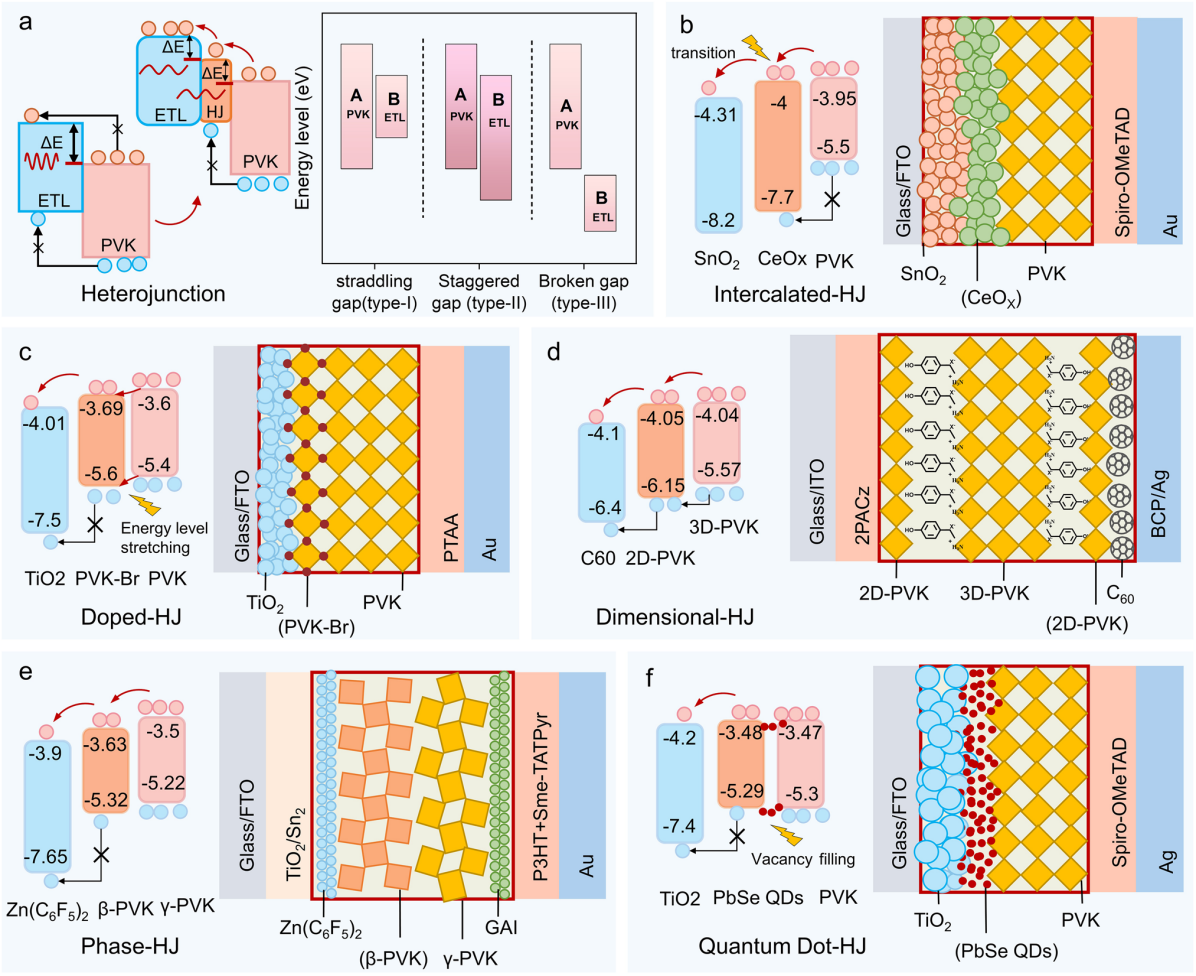
Heterojunction Strategies. **a** Working principle of heterojunctions reducing energy loss. **b** Structure and energy-level changes of intercalated heterojunctions [72]. **c** Structure and energy-level changes of doped heterojunctions [73]. **d** Structure and energy-level changes of heterojunctions with different dimensionalities [74]. **e** Structure and energy-level changes of heterojunctions with different phases [31]. **f** Structure and energy-level changes of quantum dot heterojunctions [75]

A heterojunction is an interface region formed between two different semiconductor materials. The matching degree depends on the crystal structures, similar atomic spacing, and thermal expansion coefficients of the two semiconductors. It can also be used to fabricate various semiconductor devices. As shown in Fig. 5a, heterojunction engineering reduces energy transfer losses by creating energy-level steps. Based on energy band alignments, traditional heterojunctions are classified into three types: straddling gap, staggered gap, and broken gap [76]. Among them, the staggered gap type is more conducive to electron–hole separation. The staggered heterojunction structure can be achieved via doping, material intercalation, anisotropic heterojunctions, phase heterojunctions, quantum dot heterojunctions, etc. The discussion below focuses only on the band tuning relationship of planar heterojunctions.

#### Intercalated Heterojunction

In 2009, the Miyasaka team first applied perovskite (CH_3_NH_3_PbI_3_, CH_3_NH_3_PbBr_3_) materials as photosensitizers in dye-sensitized solar cells [77]. In 2012, Park et al. introduced solid-state hole transport material (HTM) (spiro-2,2′,7,7′-Tetrakis(N,N-di-p-methoxyphenylamine)-9,9′-spirobifluorene (Spiro-OMeTAD)) for the first time, increasing the efficiency of perovskite solar cells from 3.8% to 9.7%, which officially clarified the roles of HTL and ETL in optimizing the transport and separation of charge carriers [78]. When certain ETMs exhibit excellent lattice matching or charge transport capabilities but do not meet the energy-level matching requirements of perovskite/ETL, double-layer and multi-layer heterostructures effectively address this issue, demonstrating significant performance advantages. The principle of multi-layer heterojunctions lies in leveraging the energy band gap differences of materials to insert intermediate layers into energy-mismatched materials, thereby forming stepped energy levels that reduce electron transfer barriers and enhance charge transfer capability.

In 2019, Nan et al. used a low-temperature solution method to prepare TiO_2_/SnO_2_ dual-electron-layer PSCs, achieving a PCE of 19.11% [79]. In 2021, Huang et al. inserted Nb_2_O_5_ as a step material into ZnO/perovskite, which not only served as an energy step but also prevented the detrimental effect of ZnO in acidic environments on the organic–inorganic hybrid perovskite, achieving a PCE of 13.8% for ZnO as an ETM [80]. In 2024, Liu et al. introduced a CeO_X_ interlayer into SnO_2_/PVK, demonstrating the advantage of energy-level stepping in multi-layer heterojunctions. This interlayer was a key technology in achieving a high-efficiency solar cell with a PCE of 24.63% (Fig. 5b) [72]. A series of experiments have demonstrated that using materials with different energy levels as transition layers can reduce energy losses during the electron transport process, thereby improving conversion efficiency.

#### Doped Heterojunction

The P–N junction serves as the core of cell technology, functioning through the diffusion and field effect of electrons. Due to the bipolar nature of PSCs and the inherent energy difference within their structure, a P–N junction is not essential in the perovskite structure. However, the appropriate introduction of a P–N junction can enhance the rapid separation and transport of charge carriers with minimal structural changes. PVK to ETL is used for electron transport and requires doping with a donor to regulate the process. Halogens (such as Br^−^, Cl^−^) are commonly used to achieve n-type doping of perovskite, with relatively little impact on crystallinity.

In 2024, Mike et al. used reaction force fields (ReaxFF) to perform molecular dynamic simulations, demonstrating that replacing 1/4 of the I in inorganic perovskite CsPbI_3_ with Br can improve the crystal quality through stress modulation [81]. The doping of perovskite/ETL forms a heterojunction at the interface, adjusting the matching energy levels toward a staggered type, as shown in Fig. 5c. In 2018, Hui doped Br into CsPbI_3_ within the CsPbI_3_/TiO_2_ structure, forming CsPbI_2_Br. At the quantum dot interface of CsPbBrI_2_/ CsPbI_3_, a heterojunction with a graded bandgap was formed, reducing the energy barrier in the electron transfer path between the interfaces [73]. In 2019, Man et al. used DFT simulations to introduce TiO_2_ into the CsPbI_3_/ZnO structure, proving that the addition of TiO_2_ can modulate the edge of the ZnO interface, thereby achieving energy band alignment at the interface [43]. Doping heterojunctions optimize the energy-level arrangement by establishing P-N junctions or stabilizing the perovskite structure, while surface doping of the perovskite can stretch the interface energy levels to smaller energy gaps, thereby optimizing energy alignment and carrier transport.

#### Dimensional Heterojunction

As can be seen from the perovskite structure, the introduction of larger A-site ions can result in dimensional reduction, forming one-dimensional (1D) and two-dimensional (2D) perovskites. Low-dimensional perovskites, owing to their material discontinuity, exhibit reduced light absorption ranges, localized charge carriers, strong exciton binding energies, and poor conductivity [82]. However, the good environmental stability of low-dimensional perovskites provides a new solution for passivating the surface of three-dimensional (3D) perovskite. Research has demonstrated that 2D perovskites not only passivate the surface of 3D perovskites and eliminate surface defects but, owing to differences in bandgap widths, also function as heterojunction structures to optimize the energy-level alignment between perovskite and ETL. In 2024, Azmi et al. combined alkylamine ligands with the phosphonic acid groups in self-assembled molecules (2-(9H-Carbazol-9-yl)ethyl]phosphonic Acid (2PACz) and 4-hydroxybenzylamine (HBzA)) to create a bilayer 2D/3D heterojunction inverted perovskite solar cell, achieving a highest efficiency of 25.6%, which represents a breakthrough in the PCE of current PSCs (Fig. 5d) [74]. In this context, 2D perovskite serves as the heterojunction layer, primarily enhancing the stability of PSCs through passivation. Although its role in narrowing the energy-level gap is limited, it still preserves the staggered ladder-like energy-level structure of the heterojunction, thereby reducing energy losses.

#### Phase Heterojunction

The complex interplay of temperature, pressure, chemical composition, environmental factors, and the crystal lattice structure can lead to changes in the arrangement of ions within the lattice, resulting in the formation of different phases. The formed phases (e.g., cubic photoactive phase of FAPbI_3_ (*α*-FAPbI_3_, 390K), hexagonal non-photoactive phase (*δ*-FAPbI_3_, 293K), low-temperature photoactive tetragonal phase (*β*-FAPbI_3_, 140K), and orthorhombic phase (*γ*-FAPbI_3_, 91K)) exhibit differences in symmetry and energy states, which lead to distinct optical and electrical properties among different perovskite phases [83]. Utilizing the differences among these phases to fabricate phase heterojunctions has also become a strategy for optimizing perovskite performance. In 2022, Ji et al. fabricated a solar cell using a *γ*-CsPbI_3_/*β*-CsPbI_3_ phase heterojunction, achieving a PCE of 20.1% for all-inorganic perovskites [69]. Figure 5e illustrates the phase heterojunction principle of all-inorganic perovskite *γ*-CsPbI_3_/*β*-CsPbI_3_ with an efficiency of 21.5%, as developed by Mali [31]. Phase heterojunctions primarily improve carrier separation and transport efficiency through material differences between the distinct phases. Similar to dimensional heterojunctions, to prevent energy loss, the requirements of a staggered heterojunction must be met.

#### Quantum Dot Heterojunction

Quantum dots (QD) are semiconductor nanostructures that confine excitons in three spatial dimensions. The perovskite surface inherently contains defects, and by introducing quantum dot structures, these quantum dots can not only effectively passivate the defects on the perovskite surface but also form energy band alignment with the perovskite layer, providing an efficient carrier transport channel, reducing charge recombination, and enhancing charge transport efficiency. In 2020, Wang et al. fabricated a colloidal CsPbI_3_/PbSe quantum dot heterojunction, achieving a PCE of 13.9% and exhibiting excellent moisture resistance (Fig. 5f) [75]. Liu et al. also utilized quantum dots to further regulate energy-level alignment while incorporating a CeO_X_ heterojunction into SnO_2_/perovskite [72]. QD heterojunctions achieve gradual energy-level alignment by filling the gaps between material layers, with quantum dots serving as bridges for electron transfer. Various heterojunction structures can be synergistically applied, leveraging their complementary advantages to address different types of defects under varying conditions.

## Design of Hole Transport Layer

### Functions, Energy-Level Requirements, and Common Materials for Hole Transport Layer

The hole transport layer (HTL) serves as a critical functional layer that facilitates the efficient extraction and transport of photogenerated holes while effectively blocking electron backflow. The HTL also optimizes the interface contact between the perovskite layer and the electrode, reducing defects and improving energy-level alignment, further enhancing the open-circuit voltage and power conversion efficiency. Additionally, the HTL enhances the stability and lifetime of the device by preventing the perovskite layer from interacting with the external environment. Key attributes such as excellent film-forming ability and solution processability are crucial for the efficient integration of HTL in practical devices. thereby improving the overall performance and long-term stability of the device.

**Fig. 6:**
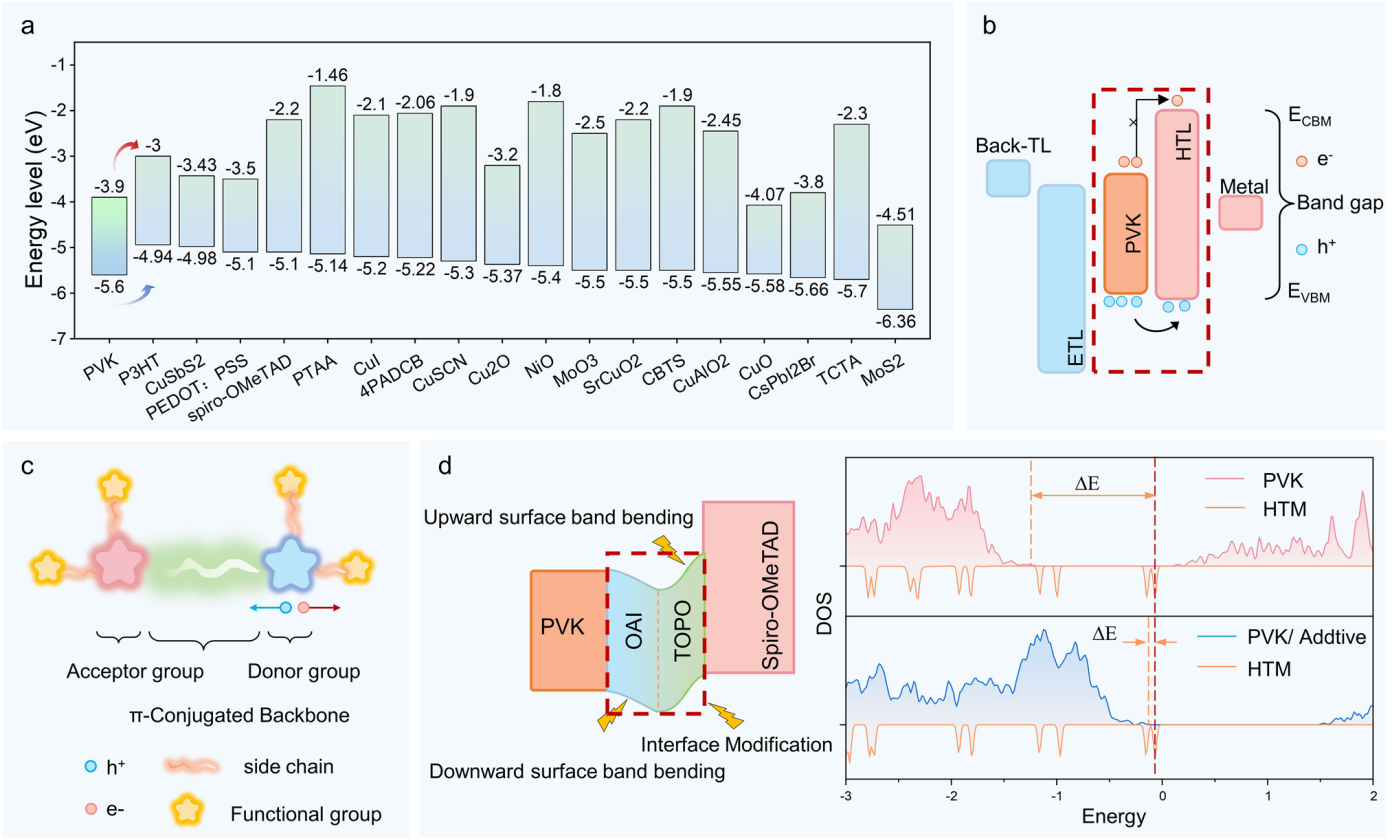
Hole Transport Layer Optimization Mechanism. **a** Energy-level alignment of hole transport materials. **b** Hole transport mechanism. **c** General structure of hole transport molecules. **d** Principle of the additive OAI and TOPO co-regulating the PVK and HTL interfaces, where the interface energy-level difference is reduced due to the influence of the additives

Figure 6a summarizes the energy-level alignment of some hole transport materials (HTMs) from the perspective of energy-level distribution. After photon excitation, the holes left by electrons in the perovskite valence band must be efficiently transferred to the HTL to minimize interfacial recombination. To achieve efficient charge separation and transport, the energy levels of the valence band of the perovskite absorber layer and the highest occupied molecular orbital (HOMO) of the organic HTM (or the valence band of the inorganic HTM) should have a small energy difference [35]. A smaller energy barrier reduces the energy loss of holes during transport. Meanwhile, the lowest unoccupied molecular orbital (LUMO) of the HTM should be significantly higher than the conduction band of the perovskite to effectively block electron injection into the hole layer, enhancing carrier separation efficiency. However, this criterion is only partially applicable. Additionally, the matching of the Fermi level is crucial as it is a key factor for carrier transport at the interface.

Hole transport materials can be classified as inorganic, organic, or organic–inorganic hybrid materials, and organic materials can be further categorized into organic polymers and organic small molecules [84]. Common organic polymer HTMs include poly(3-hexylthiophene) (P3HT), Poly(3,4-ethylenedioxythiophene):Poly(styrenesulfonate) (PEDOT:PSS), Poly[bis(4-phenyl)(2,4,6-trimethylphenyl)amine] (PTAA) and Poly[{4,8-bis[(2-ethylhexyl)oxy]benzo[1,2-b:4,5-b]dithiophene-2,6-diyl][3-fluoro-2-[(2-ethylhexyl)carbonyl]thieno[3,4-b]thiophenediyl]} (PTB7). Organic small-molecule HTMs include Spiro-OMeTAD, (4-(7H-dibenzo[c,g]carbazol7-yl)butyl)phosphonic acid (4PADCB), and tris(4-carbazoyl-9-ylphenyl)amine (TCTA). Inorganic HTMs include CuSbS_2_, opper barium tin sulfide (CBTS), CuSCN, CuAlO_2_, Cu_X_O, CuI, NiO_X_, NiCo_2_O_4_, MoO_3_, SrCuO_X_, CsPbI_2_Br, MoS_2_, MoO_X_, WS_2_, and VO_X_ [51, 84–106].

Polymers, small molecules, and inorganic HTMs each have their unique advantages. Conductive polymers, due to their long conjugate structures, rapid intramolecular charge transport, and the flexibility of thin-film deposition, exhibit high charge carrier mobility. Small molecules are characterized by ease of synthesis, precise control over molecular weight, and high charge mobility due to strong intermolecular stacking. Inorganic HTMs, particularly metal oxides and organic–inorganic hybrid materials, demonstrate excellent performance and long-term stability, capable of maintaining high efficiency under harsh conditions. However, due to the high-temperature fabrication processes and the relatively low intrinsic conductivity resulting from solution engineering, only a limited number of inorganic materials have been developed into efficient HTMs. All three types of HTMs have been extensively explored for use in perovskite solar cells, contributing to the continuous optimization of photovoltaic devices.

Among them, molecular compounds play a crucial role in perovskite devices, as they can serve not only as electron or hole transport layers but also as additives to modulate the interface properties between the transport layers and the perovskite. For example, while Spiro-OMeTAD is an efficient HTM, it sometimes causes energy transfer losses due to its mismatch with the HOMO of wide-bandgap perovskites. Therefore, the energy-level alignment between the two needs to be adjusted. Additives n-octylammonium iodide (OAI) and trioctylphosphine oxide (TOPO) can stretch the surface energy levels of PVK and Spiro-OMeTAD through chemical reactions [101], reducing energy losses at the interface Fig. 6d. In addition to additives, the material energy levels can also be optimized by directly optimizing the structure of the HTM. In energy-level alignment modulation, factors such as the conjugated structure, chain length, molecular size, and the type of functional groups in the molecules all affect the energy-level arrangement. The following section will discuss in detail how adjusting the molecular HTM or structure of molecular additives can modify energy-level alignment, thereby reducing energy losses.

### General Relationship Between Molecular Structure and Energy-Level Alignment

The ability of organic molecules to conduct electricity is one of the major breakthroughs in the field of organic chemistry. The use of organic materials as HTM has been proven to exhibit excellent charge carrier transport properties. Materials such as P3HT, Spiro-OMeTAD, and PTAA have demonstrated promising performance in current research. However, in certain scenarios, HTMs and perovskites exhibit improper matching. Current studies reveal that the primary factors influencing the energy-level alignment of molecular interface transport layers include the type of functional groups, chain length or molecular structure size, and conjugation strength. These factors can be systematically adjusted to modulate the work function, tunneling distance, or barrier height. The valence band maximum (VBM) of perovskites is approximately −5.5 eV, and the energy-level alignment can be roughly predicted based on configurational characteristics, enabling the selection of suitable HTL materials and serving as a key predictive tool in synthesizing efficient HTMs.

The general structural characteristics of undoped molecular HTMs can be traced back to the design of HTMs in dye-sensitized solar cells, typically featuring donor–acceptor (D-A) structures, *π*-conjugated frameworks, multidimensional conjugated backbones, polyaromatic structures, and functional groups (Fig. 6c) [95]. The D-A part is connected through a conjugated chain, which is key to determining the molecular HOMO–LUMO energy-level gap. By adjusting the electronic effects of the donor and acceptor, the D-*π*-A structure can precisely control the energy-level gap, thereby optimizing the energy-level alignment between the HTM and the perovskite active layer, ensuring efficient hole transport. *π*-conjugated frameworks, multidimensional conjugated backbones, and polyaromatic structures primarily enhance charge transport by increasing molecular localization and expanding charge transport pathways, thereby improving hole mobility. Functional groups (e.g., methoxy, cyano) often optimize charge transport by modulating charge density and improving the solubility and interfacial compatibility of the materials. Functional groups can also adjust the energy-level structure of HTMs to better align with the perovskite layer, reducing energy losses [94].

#### Conjugation Properties

**Fig. 7:**
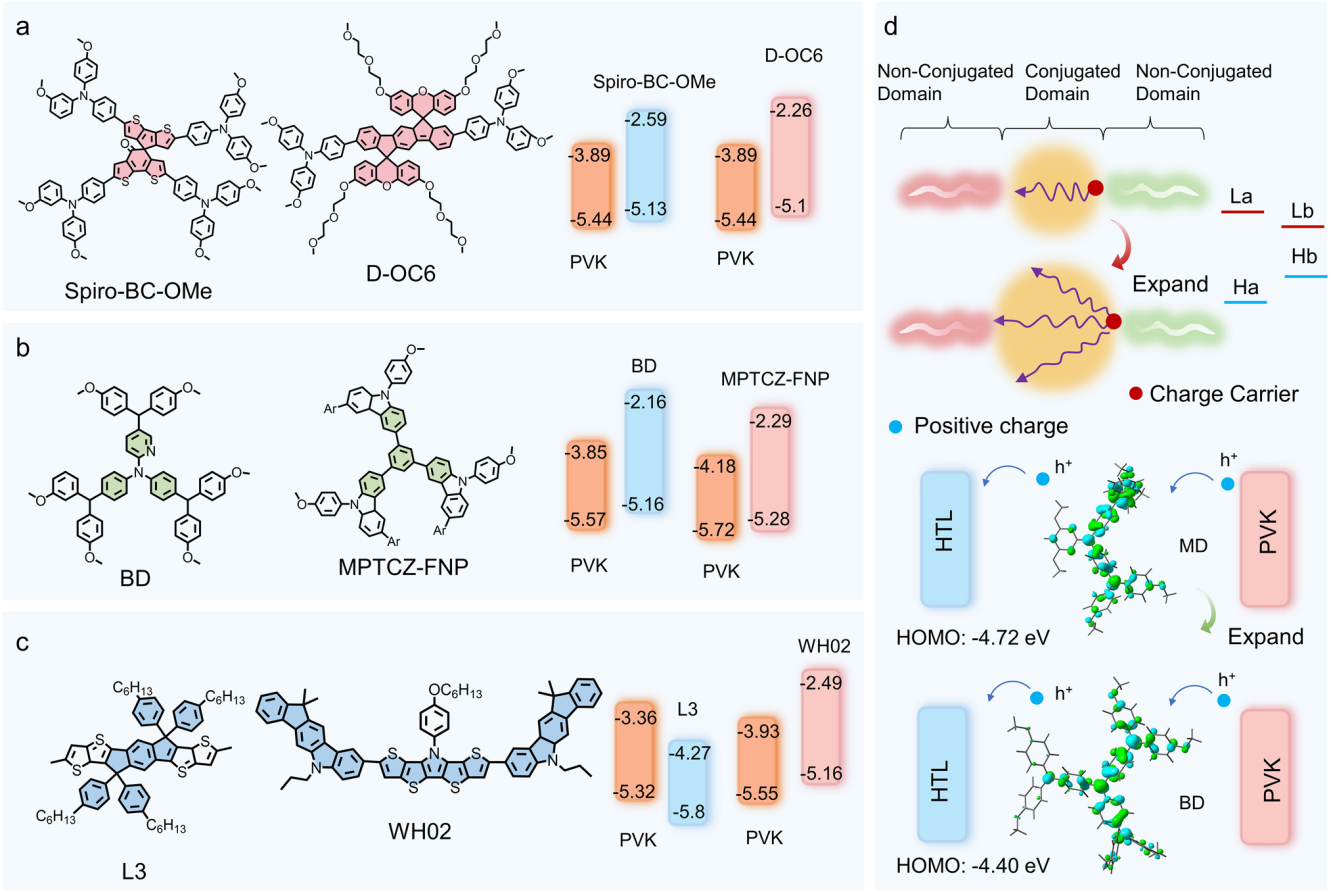
Conjugated Properties of Molecules. **a** Structure and energy-level alignment of spiral molecules [108, 109]. **b** Structure and energy-level alignment of star-shaped molecules [110, 111]. **c** Structure and energy-level alignment of linear molecules [112, 113]. **d** Principle of hole transport changes caused by conjugation

In PSCs, the conjugated structure is the core of charge transport in hole transport materials (HTMs). The overlap of *π*-electron orbitals in the conjugated structure leads to electron delocalization, which lowers the system energy, effectively enhances charge mobility and material conductivity, and reduces carrier recombination. This directly influences the overall position and energy bandwidth of the HOMO and LUMO levels (Fig. 7d). By adjusting the conjugation strength, the energy levels of HTMs can be better aligned with the perovskite active layer. The electronic and optical properties of organic conjugated molecules mainly depend on the type and length of their conjugated structures. The conjugated structures of HTMs in perovskite solar cells (PSCs) can be broadly categorized into spiro-type, star-shaped, and linear structures based on their configurations (Fig. 7a–c) [94].

Spiro-type 3D HTMs feature a non-planar three-dimensional structure and high glass transition temperature (*Tg*), which can effectively suppress molecular aggregation. Their structure consists of two *π*-conjugated fluorene units connected by an *sp*^*3*^-hybridized carbon atom, forming a vertical arrangement that enhances molecular rigidity and conjugation distribution. This design improves material thermal stability and hole transport efficiency, while optimizing the HOMO level alignment with the valence band of the perovskite, facilitating efficient hole extraction and transport. A typical spiral-type HTM is Spiro-OMeTAD. The conjugated structure of Spiro-OMeTAD is composed of two N,N′-diphenyl-N,N′-bis(3-methylphenyl)-1,1′-biphenyl-4,4′-diamine (TPD) units. By optimizing the spiral structure of Spiro-OMeTAD, other HTMs with distinct performance characteristics can be developed. For example, as shown in Fig. 7a, Spiro-BC-OMe and D-OC6, the HOMO and conduction band energy differences between these two spiral materials and perovskite are *ΔE* = 0.31 and 0.34 eV, respectively, both of which show good energy-level matching with PVK, achieving optimal efficiencies of 22.15% and 24.80%, while also demonstrating superior stability compared to traditional Spiro-OMeTAD [108, 109].

Star-shaped HTMs extend branches outward from a central conjugated unit, forming a highly symmetrical molecular structure that further expands the *π*-conjugated system, particularly in typical star-shaped HTMs composed of a single core with three or four branches. This structure not only enhances hole transport efficiency and the solubility of the material but also promotes good uniformity in the thin film, effectively preventing molecular aggregation. It represents a low-cost, high-efficiency HTM. Its high conjugation helps regulate the HOMO and LUMO energy levels, optimize interface matching, and improve photoelectric performance. Common star-shaped cores include triphenylamine (TPA), fused carbazole rings (DCZ), triazole-based derivatives (TAT), and tetraphenylethylene (TPE), among others. As shown in Fig. 7b, the star-shaped HTMs, BD with a triphenylamine core unit and 6,6′,6''-(benzene-1,3,5-triyl)tris(N-(9,9-dimethyl-9H-fluoren-2-yl)-N,9-bis(4-methoxyphenyl)-9H-carbazol-3-amine) (MPTCZ-FNP) with a triphenyl core unit, have energy differences with the perovskite conduction band of *ΔE* = 0.47 and 0.44 eV, respectively, achieving maximum efficiencies of 19.19% (all-inorganic, undoped) and 20.27% [110, 111].

Linear HTM structures (e.g., D-A, D-*π*-A, and D-A-D types, D represents an electron-rich donor, while A represents an electron-deficient acceptor) achieve efficient hole transport through one-dimensional conjugated chains, D-A groups will be discussed in detail in the next section. The length of the conjugated chain can be adjusted through molecular design to optimize the HOMO and LUMO energy levels and enhance transport efficiency. The extension of the conjugated chain increases the delocalization range of *π*-electrons, significantly improving the material's conductivity and hole mobility. For perovskite solar cell applications, the rational selection and design of HTM backbones and their conjugated characteristics can markedly enhance the device's photovoltaic conversion efficiency and long-term stability. As shown in Fig. 7c, the two linear structures, L3 and WH02, both use *π*-conjugation for linear extension. The energy differences with the perovskite conduction band are *ΔE* = 0.48 and 0.39 eV, respectively, achieving maximum efficiencies of 22.61% and 21% [112, 113].

Highly conjugated molecules typically exhibit enhanced *π*-electron delocalization, enabling more efficient charge transport along the molecular backbone. Increased conjugation generally reduces the bandgap, bringing the HOMO closer to the LUMO (Fig. 7d), N1-(4-(bis(4-methoxyphenyl)amino)phenyl)-N1-(4,6-dimethoxypyrimidine-2-yl)-N4,N4-bis(4-methoxyphenyl)benzene-1,4-diamine (BD) is a hole transport molecule that adds phenyl rings to the N2,N2-bis(4-(bis(4-methoxyphenyl)amino)phenyl)-N5,N5-bis(4-methoxyphenyl)pyridine-2,5-diamine (MD) structure, thereby expanding the conjugated core) [114–118]. By tuning the conjugation strength of HTM molecules, the energy-level alignment with other materials can be optimized.

#### Functional Groups

In the design of hole transport materials (HTMs) for PSCs, the rational selection and arrangement of functional groups are critical for energy-level modulation and charge transport performance. The functional groups in HTMs mainly include donor groups, acceptor groups, substituents, and anchoring groups. Donor groups enhance the HOMO energy level through electron-donating effects, facilitating hole transport, while acceptor groups lower the LUMO energy level by attracting electrons, preventing electron backflow and charge recombination. These groups are central to controlling the molecular energy levels. Substituents can fine-tune the HOMO and LUMO energy levels through electron-donating or electron-withdrawing effects, while also optimizing the material's solubility and film-forming properties, improving interfacial characteristics. Anchoring groups, through chemical or physical adsorption, bind to the perovskite or electrode interface, reducing interfacial defects and enhancing device long-term stability and charge separation efficiency. Rational design of these functional groups can significantly optimize the energy-level alignment and charge transport performance of HTMs, thereby improving the photovoltaic conversion efficiency and stability of PSCs [89, 119–131].

##### Donor–Acceptor Groups

D-*π*-A is a well-known molecular HTM motif. The donor–acceptor (D-A) design introduces a polarization effect through the connections at both ends of the molecule, thereby enhancing charge transfer capability and continuously reducing the HOMO–LUMO bandgap. The D-A configuration has evolved into linear HTMs such as D-*π*-A, D-A-D, and D-A-*π*-A-D. The type and electronic properties of D-A groups are key factors in determining the performance of conductive molecules. D-A polarization and stability are critical factors for optimizing HTM performance. Stronger donor groups result in higher HOMO energy levels, while stronger acceptor groups lead to lower LUMO energy levels. By combining different D and A groups, the HOMO energy level and hole transport properties of linear molecules can be flexibly tuned. Common electron-rich donor groups used in HTMs for PSCs include triphenylamine, thiophene, fluorene, and carbazole, while electron-deficient acceptor groups include triazine groups, cyano-functionalized aromatic rings, and condensed rings containing amide or imide groups.

**Fig. 8:**
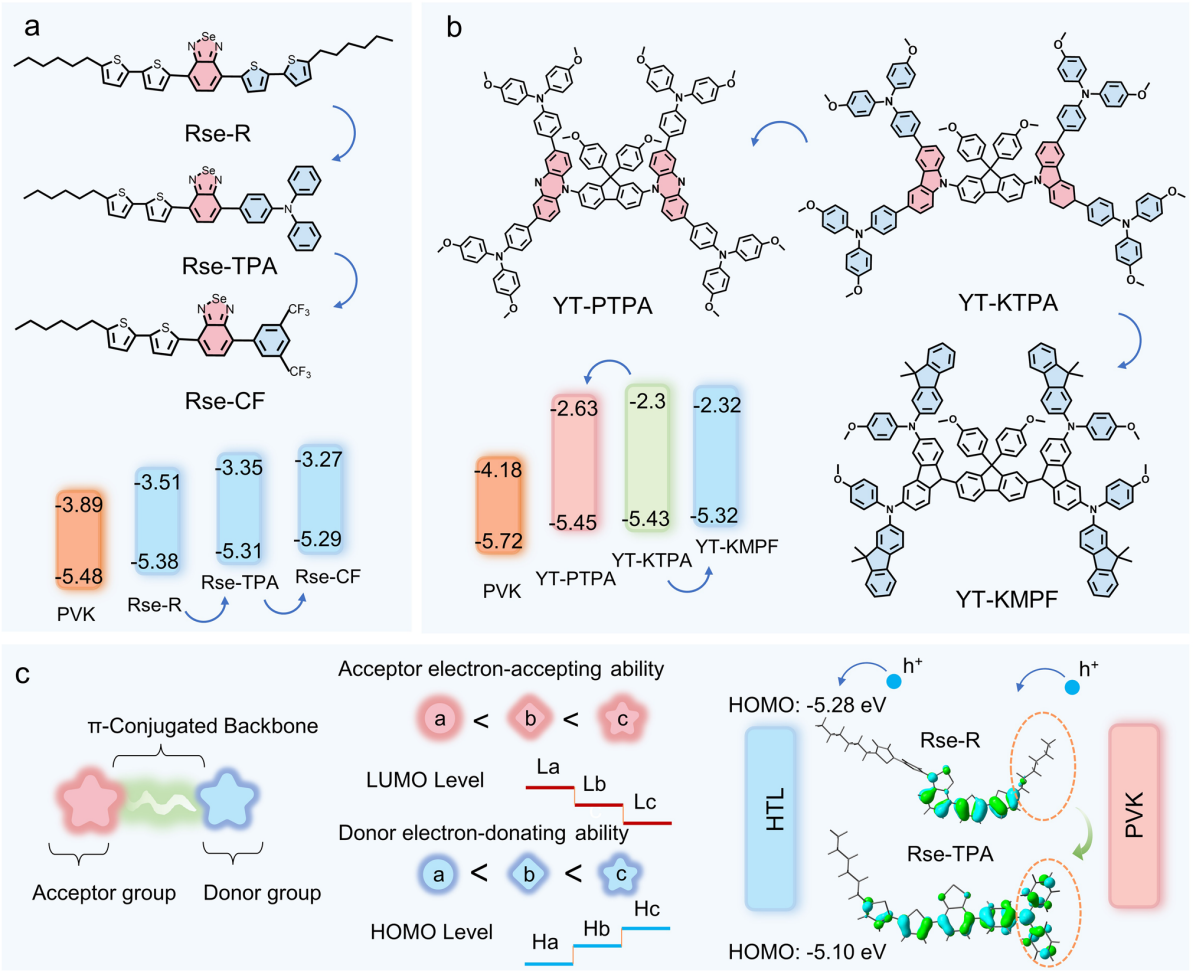
D-A Group Properties. **a** Comparison of different donors: molecular structures and energy-level changes of Rse-R, Rse-CF, and Rse-TPA [132]. **b** Comparison of different donor–acceptor pairs: molecular structures and energy-level changes of YT-PTPA, YT-KTPA, and YT-KMPF [133]. **c** Mechanism of how D-A group influences energy-level changes

D-A interactions dominate the changes in molecular energy-level alignment. Abdullah et al. demonstrated that introducing alkylthiophene (R), triphenylamine (TPA), and 3,5-bis(trifluoromethyl)benzene (CF) as donor groups into HTMs based on benzoselenadiazole and alkylthiophene acceptor units resulted in the synthesis of three HTMs: RSe-CF, RSe-TPA, and RSe-R. Variations in the donor groups significantly adjusted the HOMO energy-level differences of the molecules, thereby exhibiting excellent optoelectronic and thermal stability. As shown in Fig. 8a, donor–acceptor unit HTMs synthesized with different groups demonstrated lower HOMO energy levels. For the same acceptor, the stronger the electron-donating ability of the donor, the shallower the HOMO energy level. This makes it easier to select materials that better match the perovskite valence band, thereby improving the open-circuit voltage (*V*_oc_) and power conversion efficiency (PCE) [132].

Zhan designed and synthesized HTMs using the spiral-structured 9,9-bis(4-methoxyphenyl)-9H-fluorene unit as a *π*-bridge building block, with butterfly-shaped phenothiazine and fully planar carbazole as donor arms. At the terminal positions of the HTM, Zhan introduced traditional symmetric structures like 4,4′-dimethoxytriphenylamine and novel asymmetric structures such as N-(4-methoxyphenyl)-9,9-dimethyl-9H-fluorene-2-amine. As shown in Fig. 8b, for the same donor but different acceptors, the stronger the electron-accepting ability of the acceptor, the deeper the LUMO energy level, while the HOMO energy level changes little. On the other hand, for the same acceptor but different donors, the stronger the electron-donating ability of the donor, the shallower the HOMO energy level, while the LUMO energy level remains nearly unchanged. The HOMO levels formed by different donor units were −5.45, −5.43, and −5.32 eV, with the HOMO energy-level gaps between the HTMs and the perovskite valence band ranging from 0.27 to 0.4 eV, ensuring sufficient driving force for hole transport [133]. In conclusion, in general, the molecular energy levels change with the strength of the D-A group (Fig. 8c). Selecting appropriate donor and acceptor groups is key to optimizing the energy-level matching between HTMs and perovskites.

##### Substituent Groups

Substituent groups are functional groups attached to the backbone framework, and they typically modulate the HOMO and LUMO energy levels through inductive and conjugative effects. Although their influence on energy-level tuning is not as significant as that of D-A groups, substituents can effectively optimize the material's solubility and film-forming properties, while also improving the interface characteristics, leading to better energy-level alignment (Fig. 8d). Electron-donating substituents include methoxy (–OCH_3_), amino (–NH_2_), and alkyl (-R) groups, while electron-withdrawing substituents include cyano (–CN), carbonyl (–C = O), and halogens (–F, –Cl). The type, position, and quantity of substituent groups significantly impact electronic properties and energy-level alignment. By adjusting these factors, better energy-level matching between the HTM and perovskite can be achieved.

**Fig. 9:**
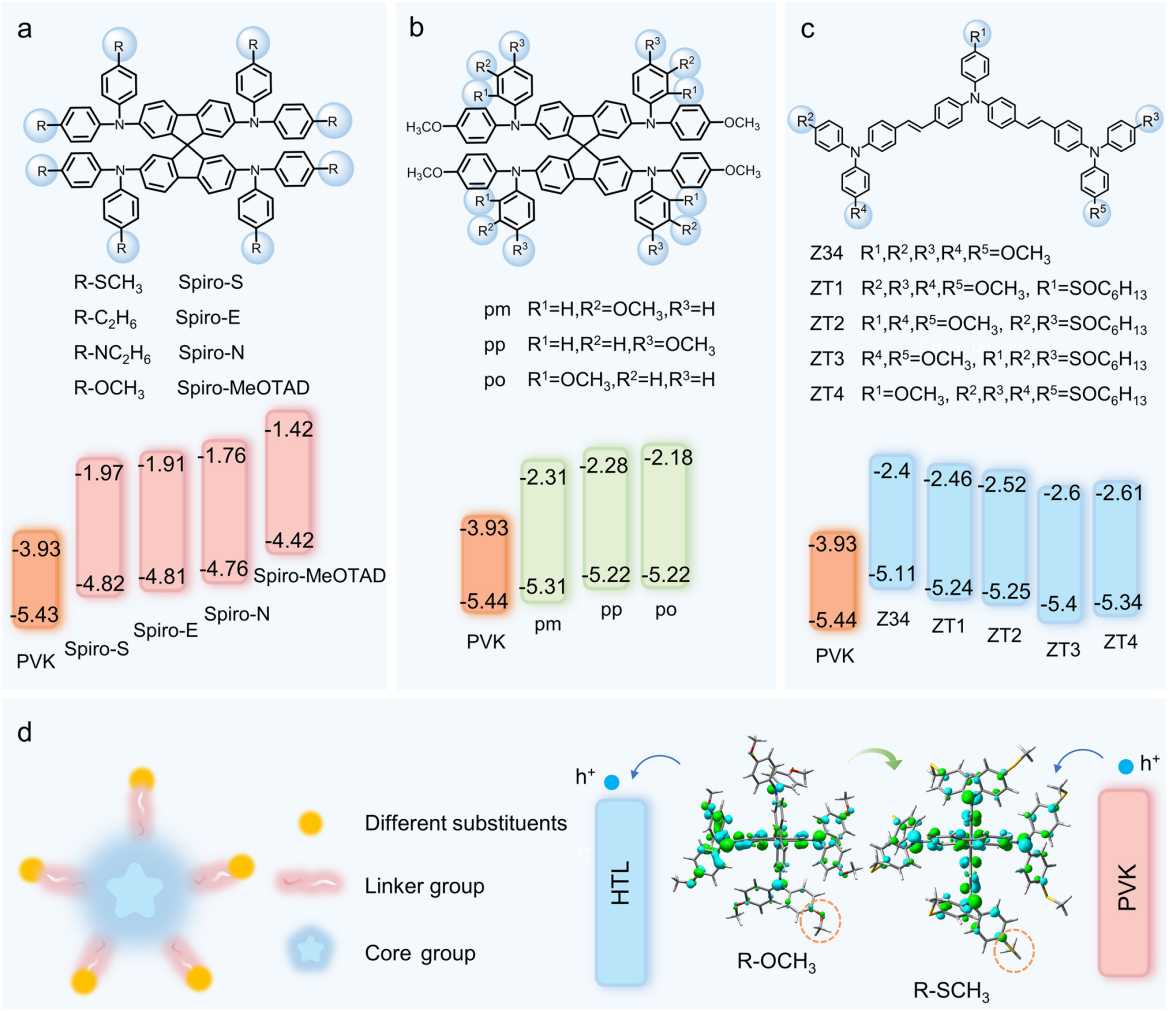
Substituent Properties. **a** Structure and energy-level changes of different substituent groups: Spiro-MeOTAD, Spiro-E, Spiro-N, Spiro-S [134]. **b** Structure and energy-level changes of different substituent positions: pp, pm, po [135]. **c** Structure and energy-level changes of different numbers of substituent groups: Z34, ZT1, ZT2, ZT3, ZT4 [136]. **d** Principle of how substituent groups influence energy-level changes

In a study by Hu, methylthio (Spiro-S), N,N-dimethylamino (Spiro-N), and ethyl (Spiro-E) groups were used to substitute the para-methoxy groups in 2,2′,7,7′-tetrakis(N,N-di-p-methoxyphenylamine)-9,9′-spirobifluorene (Spiro-MeOTAD), resulting in three new spirobifluorene-based HTMs [134]. As shown in Fig. 9a, the three substituents caused changes in molecular energy levels. The strong electron-donating ability of the N,N-dimethylamino group led to a higher HOMO level for Spiro-N, while the HOMO levels of methylthio and ethyl-substituted derivatives decreased by 0.16 and 0.05 eV, respectively, indicating the impact of substituent types on electronic properties. The authors synthesized three HTMs with better energy-level alignment by substituting different groups on the basis of Spiro-OMeTAD, further demonstrating the impact of substituent types on energy-level regulation.

In another study, Jeon systematically altered the position of -OMe substituents in spiro-type arylamine HTMs, transitioning from para to meta- and ortho-positions, to explore the structure-performance relationship in perovskite-based hybrid solar cells [135]. As illustrated in Fig. 9b, the electronic effects of spiro derivatives varied significantly depending on the substituent positions. Compared to para-substitution, meta-substitution reduced the HOMO energy by approximately 0.09 eV due to electron-withdrawing effects, while ortho-substitution increased the LUMO energy by about 0.1 eV due to increased dihedral angles. This research highlighted the impact of substituent position changes on energy-level alignment.

Additionally, Zhang investigated the effect of alkyl sulfone substituent incorporation on the structure and electronic properties of HTMs by replacing the terminal methoxy groups (-OCH_3_) of 4-methoxytriphenylamine (MeOTPA) [136]. As shown in Fig. 9c, the introduction of alkyl sulfone groups reduced molecular conjugation, leading to deeper HOMO levels and larger band gaps. When three methoxy groups were replaced with alkyl sulfone groups, the HOMO level decreased most significantly, by approximately 0.29 eV [137], validating the effect of the number of substituents on energy-level regulation. In summary, while the impact of substituents on the molecular bandgap is relatively small, the type, number, and position of the substituents all play a role in modulating the molecular energy levels, thus optimizing the energy-level alignment between PVK and HTM.

##### Anchoring Groups

**Fig. 10:**
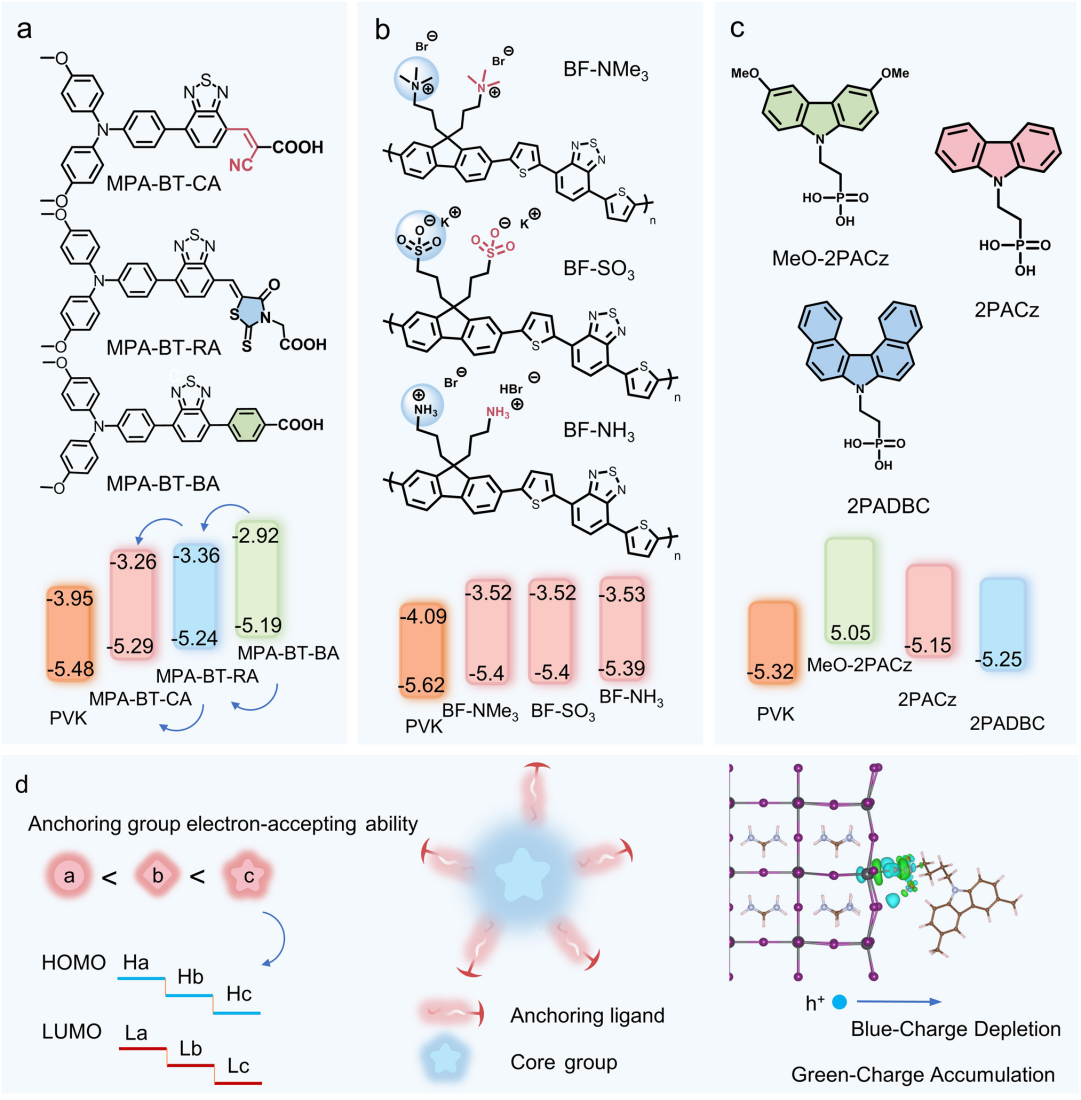
Anchoring Group Properties. **a** Structure and energy-level changes of different anchoring group types: MPA-BT-CA, MPA-BT-BA, MPA-BT-RA [139]. **b** Anchoring groups in ionic additives: structure and energy-level alignment of BF-NMe3, BF-SO3, BF-NH3 [140]. **c** Anchoring groups of molecular additives in inorganic HTMs: structure and energy-level alignment of 2PAC, MeO-2PAC, 2PADBC [141]. **d** Principle of anchoring group functionality

Anchor groups are chemical groups capable of forming strong bonds with material surfaces or interfaces, typically used to enhance interface stability or facilitate charge transfer. While anchor groups have a limited direct impact on the molecular energy levels, they primarily interact with perovskites through chemical bonds or group interactions to modify the physical and chemical properties of target molecules. This interaction reduces interfacial resistance, induces energy-level bending at the interface, optimizes energy-level alignment, minimizes non-radiative recombination at the interface, and reduces charge transport losses (Fig. 10d). Common anchor groups in HTMs include thiols, carboxylic acids, phosphonic acids, silanes, and boronic acids, which are often attached to electron-rich aromatic structures like carbazoles, triphenylamines, and phenothiazines at the HTM termini. The type of anchor group can influence interfacial dipoles, recombination energy losses, contact resistance, coverage, and work function of the substrate [138].

Liao et al. designed and synthesized a series of low-cost D-A type HTMs (MPA-BT-CA, MPA-BT-BA, and MPA-BT-RA) by incorporating anchor groups such as 2-cyanoacrylic acid (CA), benzoic acid (BA), and rhodanine-3-propionic acid (RA) into the main structure of 4-methoxy-N-(4-methoxyphenyl)-N-phenylamine (MPA) and benzothiadiazole (BT). The MPA-BT-CA and MPA-BT-BA modified ITO substrates exhibit higher work functions, which are beneficial for improving *V*_oc_ in the corresponding perovskite devices. The PSCs fabricated with MPA-BT-CA not only achieve 21.81% efficiency but also exhibit good stability [139]. As shown in Fig. 10a, the molecular energy-level changes of the three anchor groups in the HTMs are minimal, with the HOMO energy difference *ΔE* within 0.1. The electron-withdrawing ability of the CA and RA groups is stronger than that of the BA group. Electron-withdrawing groups attract electrons from the molecule, reducing the electron density within the molecule, thus lowering both the HOMO and LUMO energy levels.

Ion additives are commonly used as interface additives to anchor the interface or transport layers [127, 131, 142]. As materials for interface engineering, these additives act as a bridge between interfaces, especially for inorganic crystalline materials with fixed lattice arrangements. They passivate surfaces and facilitate electron transfer, thereby improving crystallinity and reducing interfacial resistance. Groups that interact with perovskites or ETMs/HTMs can also function as anchor groups.

Liu et al. synthesized three conjugated polyelectrolytes (CPEs) with a conjugated backbone composed of 4,7-bis(thiophen-2-yl)-benzothiadiazole and fluorene units, and ionic anchor groups including -N(CH_3_)_3_^+^, -SO_3_^−^, and -NH_3_^+^. These CPEs (BF-NMe_3_, BF-SO_3_, BF-NH_3_), used in conjunction with PEDOT:PSS as HTMs, demonstrated improved interface properties. As shown in Fig. 10b, the three CPEs share similar HOMO levels (~ − 5.40 eV), which are deeper than that of PEDOT:PSS (− 5.20 eV). This indicates that ionic anchor groups have a relatively weak impact on energy levels but effectively enhance energy-level alignment and interfacial connectivity [140].

Molecular additives exhibit similar regulating characteristics. Li et al. investigated the root cause of performance loss in Sn-based PSCs using NiO_X_ as the HTL and found that nickel cation defects at the perovskite–NiO_X_ interface act as Lewis acids and oxidizing agents, leading to Sn^2^⁺ oxidation and creating barriers to hole extraction. By introducing the 2PADBC molecule, which contains phosphonic acid groups as anchor groups, they passivated nickel cation defects and reduced interfacial non-radiative recombination caused by tetravalent tin defects. As shown in Fig. 10c, the study compared three Lewis acids (2PACz, MeO-2PACz, 2PADBC) with carbazole as the core unit and phosphonic acid groups anchoring to nickel cations. The modified NiO_X_ exhibited better energy-level alignment with perovskites, reducing interfacial transport barriers. Among them, 2PADBC demonstrated the best-matched valence band maximum (VBM), leading to an increase in *V*_oc_ from 0.712 to 0.825 V [141].

#### Chain Length and Structural Size

The molecular size is a determining factor in the selection of HTMs. Longer molecular chains can enhance charge transport efficiency by promoting charge transmission through extended conjugated systems, thereby improving the performance of optoelectronic devices. Additionally, longer molecular chains can broaden the light absorption range of the material, increasing light-harvesting efficiency. However, excessively long chains may disrupt molecular stacking arrangements, increasing transport barriers and adversely affecting effective charge transport. Larger molecular structures contribute to optimizing energy-level alignment, improving mechanical strength and thermal stability, and enhancing the optical properties of the material. Overall, appropriately tuning the chain length and molecular structure is crucial for optimizing the performance of HTMs in perovskite photovoltaic devices. Long-chain polymers exhibit higher charge transport efficiency and better film-forming properties, while small-molecule HTMs demonstrate excellent charge transport performance through tight molecular packing and flexible energy-level tuning. In the functional structure of molecular materials, the conjugated backbone is a core factor for charge transport. Apart from the structural characteristics of the backbone influencing molecular energy-level alignment, adjusting the backbone chain length can effectively regulate molecular energy levels, achieving energy-level alignment between the HTM and the perovskite interface. This design strategy has been widely applied. However, the side chains of HTMs have minimal influence on molecular energy levels and are primarily used to improve the thermal stability and surface smoothness of the films, as well as for interface modification [143–146].

In the D-*π*-A structure, the *π*-conjugated backbone primarily serves as the charge transport pathway, determining the molecule's ability to facilitate charge spatial transfer. Although *π*-conjugated backbone is not the primary factor influencing energy-level alignment, its chain length and size can modulate the polarization effects of the D-A structure, thereby indirectly affecting the energy alignment properties of the material.

**Fig. 11:**
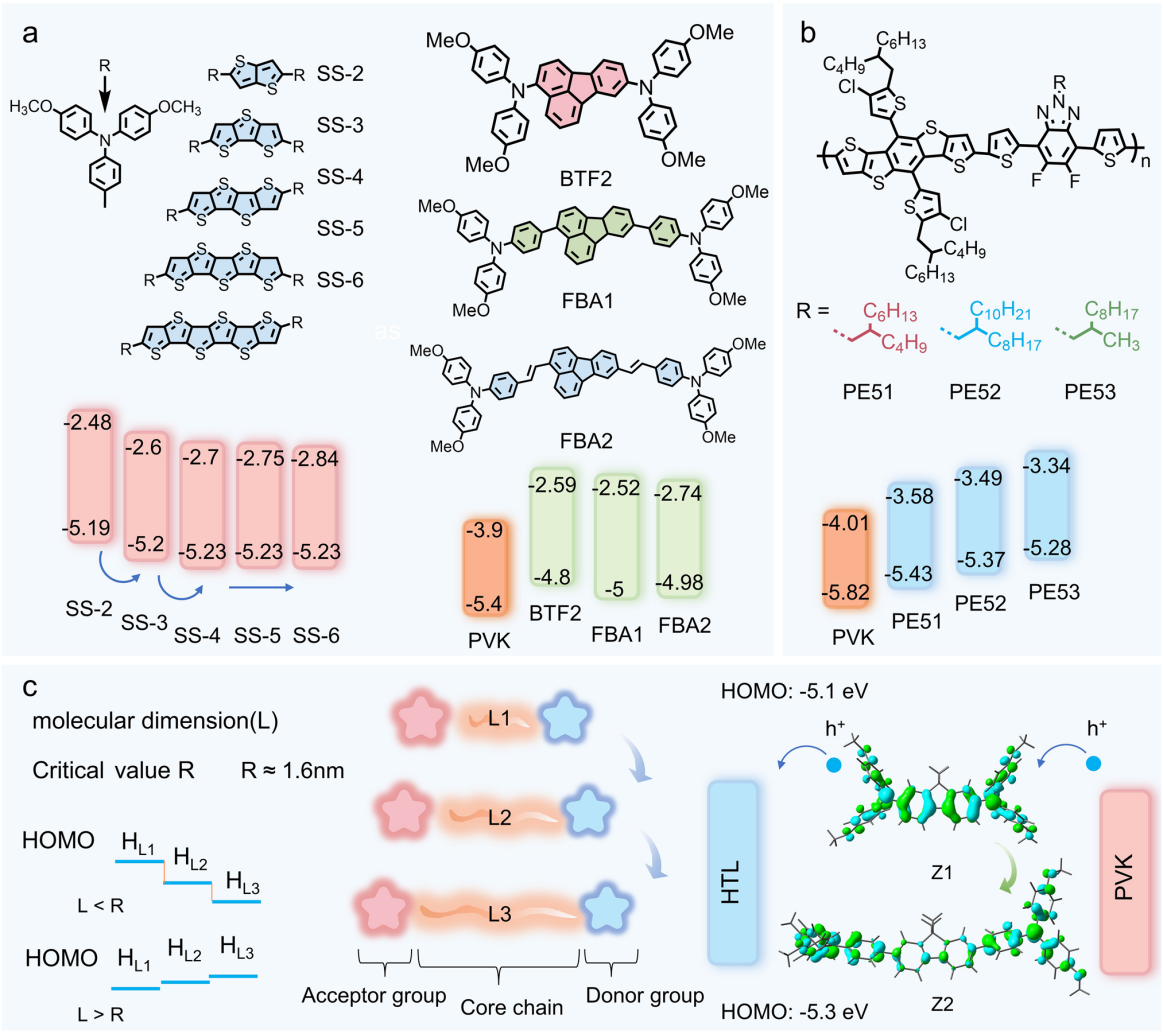
Molecular Chain Size Properties. **a** Structure and energy-level changes of different backbone lengths: SS-2, SS-3, SS-4, SS-5, SS-6 [103]. Structure and energy-level changes of different backbone chain lengths: BTF2, FBA1, FBA2 [147]. **b** Structural and energy-level variations with different side chain lengths: PE51, PE52, PE53 [148]. **c** Mechanism of how chain length influences energy levels, Structural and energy-level changes of different molecular lengths: Z1, Z2 [149]

To investigate the impact of chain length on molecular materials, Wei et al. examined the thiophene backbone chain length in D-*π*-D HTMs to identify optimal structural parameters. As shown in Fig. 11a, the study incorporated triphenylamine groups at both ends of the thiophene chain and varied the number of thiophene rings (*n*) to explore backbone chain characteristics. The research revealed that the LUMO level and bandgap decreased with increasing n, while the HOMO level gradually decreased with increasing n when *n* < 4. For *n* > 4, the HOMO level stabilized at approximately −5.23 eV [103]. In Duan's study, it was indicated that when using alkyl chains as quantum dots for charge extraction and transport at the PVK and C interface, an alkyl chain length of 12 carbon atoms (approximately 1.6 nm) best balances the Coulomb repulsion force and the quantum tunneling distance.

Similarly, Sun employed a fluoranthene framework to develop a series of undoped small-molecule HTMs (Fig. 11a) [147]. Using fluoranthene as the core structure, Sun synthesized BTF2. However, due to its high HOMO energy level, which caused poor alignment with the perovskite energy levels, and its low hole mobility, BTF2 was unsuitable as an efficient HTM. To address these issues, Sun modified the structure of BTF2 by attaching para-methoxy-substituted TPA units to the fluoranthene core via single bonds, forming the linear structure FBA1. Furthermore, an ethylene bridge was inserted into FBA1 to connect the TPA units with the fluoranthene core, yielding FBA2. The study showed that compared to the original BTF2 structure, the HOMO energy levels of FBA1 and FBA2 decreased by 0.2 and 0.18 eV, respectively, improving energy-level alignment with the perovskite and enhancing hole transport capabilities. And Fang replaced one fluorene arm of Spiro-OMeTAD with two methyl groups, generating a linear HTM derivative, N2,N2,N7,N7-tetra(4-methoxyphenyl)-9,9-dimethyl-9H-fluorene-2,7-diamine. Based on this structure, two phenyl groups were further introduced to synthesize 4,4′-(9,9-dimethyl-9H-fluorene-2,7-diyl)bis[N,N-bis(4-methoxyphenyl)aniline]. By extending the π-conjugated backbone, the HOMO energy level of Z1 was lowered by 0.2 eV (Fig. 11c), enabling Z2 to achieve better energy-level alignment with the valence band of the perovskite, thereby making it an optimized HTM [149]. The experiments conducted by Sun and Fang illustrate that phenyl insertion and backbone elongation are effective strategies to influence HTM energy-level alignment, providing practical insights into designing efficient HTMs.

The side chains of HTM molecules are generally not used for charge transport, but rather influence the material's solubility, stability, morphology, and compatibility with other layer materials. Modifying the position of the side chains and adding functionalized or conjugated side chains can optimize the HOMO energy level, hole extraction capability, and passivation ability of the HTM. Commonly used side chains include alkyl chains, fluorinated alkyl groups, etc. As shown in Fig. 11b, Ding, in his study of side-chain engineering of benzotriazole-based polymers as hole transport materials, investigated the effect of side chain size on hole transport by varying the side chain lengths. Increasing the length of the alkyl side chain causes the molecular stacking pattern to favor *π*-*π* stacking, which enhances the electron-withdrawing ability of benzotriazole, leading to a gradual decrease in the HOMO energy level [148].

In summary, molecules employed as charge transport layers or interfacial modifiers can effectively passivate defect sites in halide perovskite solar cells through bonding or electrostatic interactions, thereby reducing non-radiative recombination. Moreover, they can alter the interfacial properties of the perovskite crystal, enhance energy-level alignment at contact interfaces, and improve charge carrier transport efficiency, ultimately increasing the open-circuit voltage and overall device performance. These molecules also play critical roles in regulating crystallization quality and enhancing the device's environmental stability against moisture, heat, and oxygen. When used for energy-level modulation, molecular compounds can be tuned by adjusting the backbone length, donor–acceptor (D–A) strength, and the size of the conjugated segment, enabling stepwise energy-level alignment between adjacent interfaces. Meanwhile, the introduction of functional groups or variation in side chain dimensions can influence the surface energy landscape and spatial occupation of the crystal surface, allowing for energy-level stretching at the contact interface and minimizing energy offsets. Section 4.3 highlights representative case studies of molecular design strategies for improving the performance of perovskite solar cells.

### Application of Self-Assembled Molecules

Due to the structural diversity of molecules, synthesizing molecular materials tailored to specific structural requirements has become a focal point in current research. Conjugated structures influence the position of the work function and bandgap width, donor–acceptor (D-A) structures dictate molecular orbital alignment, chain length and main-chain size modulate the HOMO energy level, while side chains and functional groups optimize interfacial resistance and charge injection efficiency. These strategies collectively form the basis of current approaches to designing HTMs with energy levels compatible with PSCs. Currently, there have been many developments in the self-assembled layers for electron–hole management [106]. Molecular design strategies can be categorized into doped and undoped, single-layer and multi-layer, or ionic and molecular, all aimed at improving the quality of the transport layer while minimizing energy loss, with applications across various transport scenarios, For example, HTMs, SAMs (self-assembled monolayers), interface bridges, and doping.

**Fig. 12:**
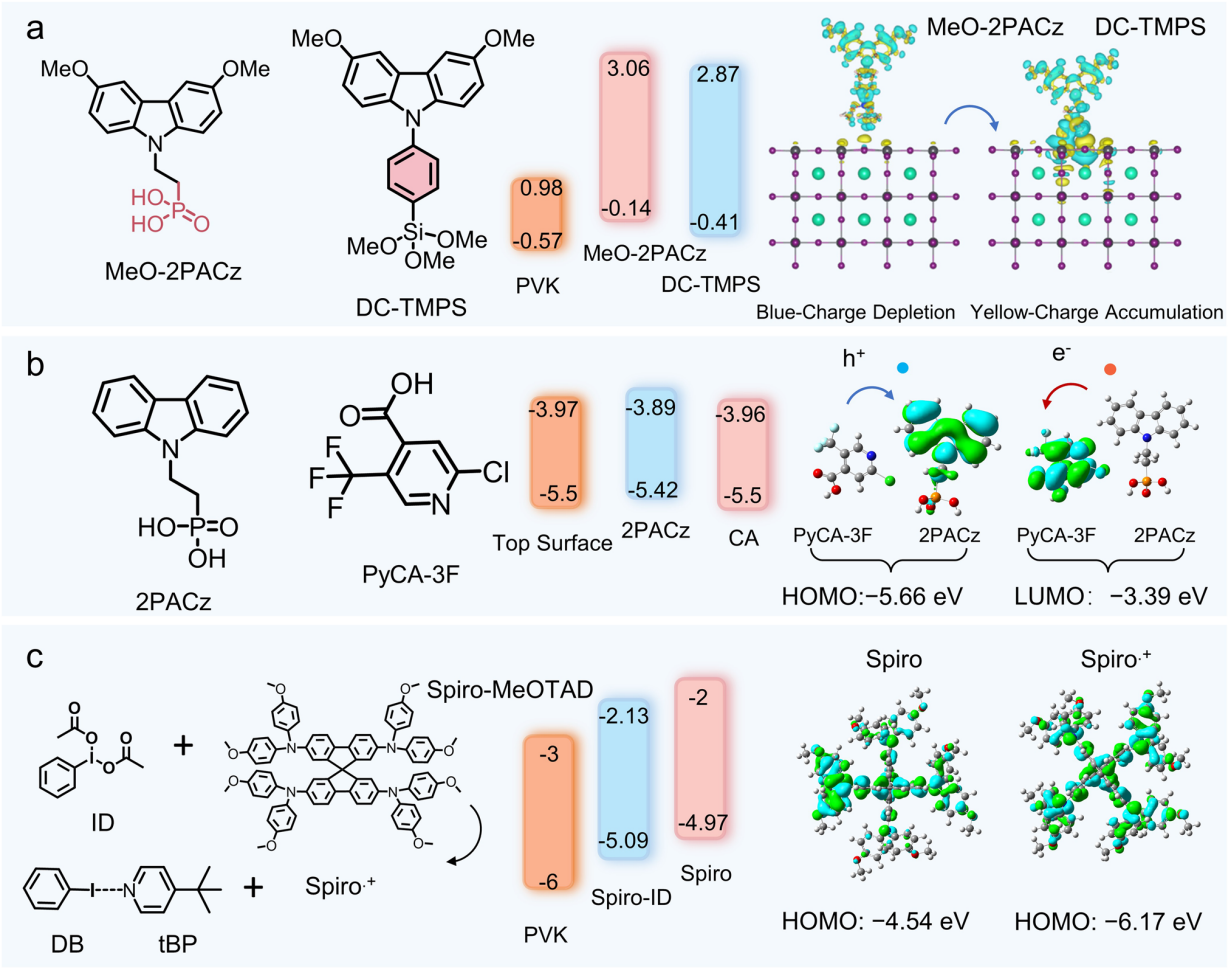
Self-assembled Molecules. **a** SAM structures and energy-level alignment of self-assembled MeO-2PACz and DC-TMPS [104]. **b** SAM structures and energy-level alignment of self-assembled 2PACz and PyCA-3F mixed SAM [150]. **c** Structural diagram of hole transport material spiro and additive design, along with energy levels of spiro before and after doping (Spiro-ID) [151]

SAM is a single-layer thin film spontaneously assembled by molecules, typically used for surface modification and interface engineering. In this structure, the molecules automatically arrange themselves into an ordered monolayer on a solid surface through non-covalent interactions (such as van der Waals forces, hydrogen bonds, electrostatic forces, etc.) or covalent bonding. Tang synthesized a SAM as a hole transport layer (HTL) for stable inverted PSCs. The SAM (DC-TMPS) features a *π*-conjugated backbone based on a triphenylamine (TPA) donor structure, incorporating silane bridge units and methoxy (-OCH_3_) substituents. The silane acts as a linkage bridge, achieving a PCE of 24.8% in the resulting PSCs (Fig. 12a) [104]. At the same time, Li employed a co-adsorption strategy using 2-chloro-5-(trifluoromethyl) pyridinecarboxylic acid (PyCA-3F) and 2PACz in his experiment, facilitating smooth surface formation and favorable energy band alignment. This approach resulted in a PCE of 24.68% (Fig. 12b) [150]. Additionally, Lan et al. introduced iodobenzene diacetate (ID) as a dopant into the Spiro-based HTL. As shown in Fig. 12c, the generated Spiro·⁺ radical species achieved optimized energy-level alignment with the perovskite absorber, substantially reducing interfacial energy losses and yielding a champion power conversion efficiency (PCE) of 25.76% [151]. Concurrently, the byproduct iodobenzene (DB) formed a halogen-bonded *t*BP-DB complex with 4-*tert*-butylpyridine (*t*BP) in the HTL through interactions, effectively suppressing chemical erosion of perovskite by *t*BP and enhancing device reproducibility.

The three researchers independently designed SAM, mixed SAM, and molecules used as HTM ion additives, all achieving high photovoltaic conversion efficiencies. This indicates that by tuning properties such as molecular chain length, functional groups, and conjugation, energy-level alignment can be controlled to obtain the desired HTM. This optimization of energy-level matching in PSCs reduces energy loss, making it a key strategy for improving device performance. Of course, molecular strategies are also applicable to the ETL.

## Summary and Outlook

The working principle of solar cells involves the absorption of light energy by specialized materials and its conversion into charge carriers, resulting in the generation of a potential difference. Understanding the properties of perovskite (PVK) materials and their energy absorption mechanisms is crucial for improving the efficiency of solar cells. This paper centers on the process of solar energy conversion into electrical current and examines the energy transfer obstacles within perovskite materials. Specifically, it reviews the energy transfer mechanisms between the perovskite light-absorbing layer and the electron–hole transport layers (HTL and ETL) in perovskite solar cells (PSCs), as well as the impact of various material designs on energy loss. Theoretically, HTLs and ETLs are expected to form a staggered band alignment with PVK. However, mismatched energy levels can be mitigated through tuning strategies such as heterojunctions, interfacial modification layers, and surface passivation to optimize material performance. This paper further summarizes the energy-level alignment of commonly used Hole-electron transport materials (ETMs and HTMs) in PSCs and discusses strategies for modifying energy levels, such as doping, phase engineering, dimensional tailoring in inorganics, and adjustments in chain length, conjugation, or functional groups in organics. Whether PSCs are single-junction or multi-junction, organic or inorganic, n-i-p or p-i-n structures, or include transport layers, the separation and transport of electrons and holes remain pivotal to enhancing device efficiency.

To continuously improve the efficiency of perovskite solar cells, several key improvements are required. First, designing perovskite materials with suitable bandgaps and energy-level alignment based on their spectral characteristics is critical for enhancing photoelectric conversion efficiency. Second, optimizing electron–hole transport layers to accelerate the movement of charge carriers is essential for improving efficiency and current quality. Finally, ensuring energy-level matching and structural stability in PSCs is vital for achieving higher conversion efficiencies. To further reduce energy losses in perovskite solar cells (PSCs), strategies such as photon up/down conversion, as well as tandem cell configurations, can be employed to broaden the spectral absorption range and overcome the limitations of conventional absorption windows. In parallel, the application of highly efficient charge transport materials combined with well-aligned energy levels can significantly minimize carrier energy dissipation during transport. Moreover, thermal energy recovery approaches (such as reverse-assisted charge separation) also offer promising pathways for enhancing the overall energy utilization efficiency.

Although various strategies have been proposed to improve light absorption, reduce energy losses, and optimize energy-level alignment, their practical adaptability remains limited. This is primarily due to the difficulty in simultaneously achieving optimal energy-level matching, charge carrier mobility, cost-effectiveness, environmental compatibility, and long-term operational stability. Additionally, factors such as film thickness and fabrication processes also influence optical properties and energy-level positioning, resulting in considerable gaps between theoretical performance and actual device outcomes. Striking a balance among these factors and overcoming related constraints represent major challenges in current PSC research. A single optimization approach is no longer sufficient to meet the escalating demands for device performance. Future research must shift toward system-level engineering methodologies that integrate multi-physical field coordination to achieve breakthroughs in PSC efficiency and stability across diverse application scenarios.

Specifically, system-level optimization should be guided by the intended application scenarios, beginning with the selection of core materials that simultaneously exhibit strong light-harvesting capabilities and efficient charge transport properties. This should be followed by precise tuning of the materials’ physical characteristics through strategies such as lattice strain engineering, hot carrier management, multiple exciton generation (MEG), and intermediate band (IB) engineering. On this basis, the integration of heterojunction engineering and additive strategies can further enhance material properties, including corrosion resistance, energy-level alignment, and defect passivation. Ultimately, a suite of comprehensive optimization approaches should be employed to maximize device performance and longevity. For instance, thermal energy recovery systems, photo/thermo-responsive passivation agents, self-healing encapsulation technologies, and chemical regeneration mechanisms—collectively contributing to the overall enhancement of operational efficiency and device lifespan.
